# Slender but Strong: Substrate-Driven Adaptations in Parasitic Wasp Ovipositors

**DOI:** 10.1093/icb/icag129

**Published:** 2026-07-23

**Authors:** Uroš Cerkvenik, Robin Heinen, Johan L van Leeuwen, Sander W S Gussekloo

**Affiliations:** Department of Biology, University of Ljubljana, 1000, Ljubljana, Slovenia; Terrestrial Ecology Research Group, Department for Life Science Systems, Technical University of Munich, D-85354, Freising, Germany; Experimental Zoology Group, Wageningen University and Research, 6708 WD, Wageningen, The Netherlands; Experimental Zoology Group, Wageningen University and Research, 6708 WD, Wageningen, The Netherlands

## Abstract

The hymenopteran ovipositor is a sophisticated probing organ composed of three sliding elements, interconnected with two tongue-and-groove (olistheter) mechanisms. The slender ovipositor must accommodate at least two mechanical requirements: the strength to puncture a substrate and the flexibility to navigate through it. During puncturing, the ovipositor is often mechanically stabilized with external supports, while coordinated pushing and pulling of the three ovipositor elements generate motions through the substrate. Both mechanisms reduce bending moments along the ovipositor thereby preventing failure-prone buckling deformations. Despite the efficacy of these mechanisms, it is anticipated that drilling into stiff and tough media requires reinforced structures, creating a potential trade-off with maneuverability. Here, we investigated whether ovipositor morphology reflects substrate-specific adaptations across the two largest hymenopteran families: Ichneumonidae and Braconidae. We compared 22 morphometrics measured from the ovipositor cross-sections of 86 species reported in the literature. Our results indicate that while the gross cross-sectional anatomy remains conserved across all species spanning across a wide size range, specific structural adaptations emerge at ecological extremes. We observed significant negative allometry in internal channel size, suggesting that larger ovipositors exhibit relatively thicker outer walls thereby enhancing mechanical reinforcement. Additionally, species probing tougher substrates—such as wood or (chitinous) pupae—exhibited more robust olistheters that likely prevent valve separation under high axial loads. These findings suggest a fundamental trade-off, namely, that the reinforced dorsal valve and olistheters increase flexural rigidity but limit steering capabilities. For instance, wood-boring species possess less-rigid structures than pupal parasitoids, likely to facilitate the maneuverability required to navigate complex substrates. Our results suggest that ovipositor anatomy is a finely tuned result of trade-offs between penetration force, steerability, and metabolic resource allocation. This research helps to understand hymenopteran life history and may facilitate bio-inspired design of steerable surgical needles.

## Introduction

Parasitoid wasps are among the largest and most diverse insect lineages (Davis et al. 2010; Heraty et al. 2011; Quicke 2014; Burke and Sharanowski 2024; Hambäck et al. 2024). This diversity is attributed to their parasitic lifestyle and co-evolution with their predominantly herbivorous hosts (Davis et al. 2010; Peters et al. 2017; Blaimer et al. 2023; Cruaud et al. 2023). Plant tissues, especially their chemical composition and material characteristics, seem to be an important driver of hymenopteran speciation as these affect both the parasitoids and their hosts.

Parasitic wasps’ adaptation is steered by plant material characteristics because many of the species use an ovipositor to probe (plant) material and locate the hosts hidden within (Nenon et al. 1997; Brown and Anderson 1998; Quicke et al. 2000; Dweck et al. 2008; Ghara et al. 2011; Kundanati and Gundiah 2014; Cerkvenik et al. 2017). After the host is found, it is often punctured with the ovipositor either to inject venom, deposit the egg(s), fluids that mark the parasitized host, and even secreting solutions that harden and build the so called “feeding tube” which allows the wasp to extract body fluids from the host (Pupedis 1978; Nufio and Papaj 2001; Vårdal 2006; Gal et al. 2014; Eggs et al. 2023). In some cases, the ovipositor is also used to secure hosts in place mechanically (van Lenteren et al. 1998; Quicke et al. 2000; Boring et al. 2009) or by injecting venoms (Edson et al. 1982; Vårdal 2006; Mitra 2013; Gnatzy et al. 2018). This makes the ovipositor an essential structure in the life history of parasitic wasps.

**Fig. 1:**
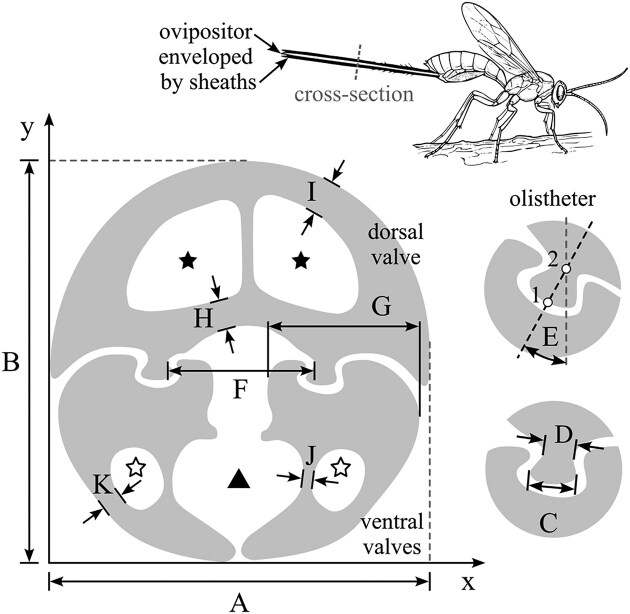
The morphometrics measured in ovipositor cross-sections. The ovipositor is generally a slender needle-like structure located at the animal’s abdomen and surrounded by sheaths. It is comprised from three valves, one dorsal and two ventral ones. Each ventral valve connects to the dorsal one with an olistheter. The olistheter consists of the tongue (rhachis) extending from the dorsal valve and a groove in the ventral valve (aulax). The valves enclose the egg canal (black triangle) and contain nerve and tracheal channels (stars). We measured the following morphometrics (see also Table 2): A—ovipositor width, B—ovipositor height, C—rhachis head width, D—rhachis neck width, E—rhachis angle obtained by first fitting a straight line (black, dashed) through the rhachis apex (point 1) and the midpoint of its neck base (point 2), and calculating its angle with respect to the vertical, dorso-ventral axis (light gray, dashed), F—distance between the rhachides, G—lateral wall thickness of the ventral valve, H—inner dorsal wall thickness, I—outer dorsal wall thickness, J—inner ventral wall thickness, and K—outer ventral wall thickness. Additionally, we measured the cross-sectional area (CSA) and computed the second moments of area in horizontal (*x*) and vertical (*y*) directions for the ovipositor and its individual valves, as well as the CSA of the channels in the dorsal valve (black stars) and ventral valves (white stars).

The ovipositor of parasitic wasps is a slender (high length-to-width ratio) tube composed of three interconnected elements, termed valves or valvulae (Fig. 1). In general, it consists of a single dorsal valve and two ventral ones. The valves are joined by two “tongue-and-groove” sliding joints known as the olistheters. The “tongues” (rhachides) are located on the dorsal valve, while the grooves (aulaces) are located on the ventral valves, allowing the valves to slide longitudinally along one another without separating (Snodgrass 1933; King 1962; Copland 1976; Dweck et al. 2008; Eggs et al. 2018; van Meer et al. 2020; Csader et al. 2021). This sliding mechanism is one of the key features that makes probing with slender ovipositors possible (Vincent and King 1995).

Puncturing a substrate with a probe as thin as the ovipositor, which are often only tens of µm in diameter or less, is difficult—such a probe is prone to bending and even breaking (buckling) when pushed into the substrate on one end. It is therefore essential for the wasp to avoid such catastrophic damage if it wants to lay its eggs. This problem has been overcome by the development of a reciprocal push–pull mechanism of the ovipositor valves. As the wasp alternatively pushes and pulls on the valves, it exploits the friction of the stationary valve(s) to anchor the organ while simultaneously pushing the other(s) forward, thereby progressing deeper into a substrate with a minimal net pushing force from the body (Vincent and King 1995; Cerkvenik et al. 2017; Cerkvenik, Dodou et al. 2019). This mechanism effectively transforms the slender organ into a “self-propelling drill,” allowing for probing without buckling, even in tough substrates such as wood (Vincent and King 1995; Parittotokkaporn et al. 2009; Cerkvenik, Dodou et al. 2019). In addition, many species also use the push-pull mechanism to steer the ovipositor through the substrate in the direction of the host, often via complex curved trajectories (Compton and Nefdt 1988; Kundanati and Gundiah 2014; Cerkvenik et al. 2017). Although the steering mechanisms are not fully understood, it has been shown for at least one species (*Diachasmimorpha longicaudata*) that alternating valve movements are used (Cerkvenik et al. 2017).

Ovipositors can be slender because the muscles that actuate the push–pull mechanism are located within the wasp’s abdomen (van Meer et al. 2020; Eggs et al. 2023). The ovipositor, therefore, only contains the egg/venom canal and hemolymph canals for tracheae and nerves that transmit sensory feedback from the tip. This morphology means that the mechanical behavior of the ovipositor—its bending and resistance—is by a large degree determined by the structural and material properties of the cuticle itself (Quicke 1991; Ghara et al. 2011; Cerkvenik, van Leeuwen et al. 2019). For example, metal atoms that are thought to reduce wear and harden the cuticle have been found in the ovipositors of parasitoids that probe in stiff substrates (Quicke et al. 1988).

While the general mechanism of ovipositor insertion has been theoretically established (Vincent and King 1995) and confirmed at least in two species (Cerkvenik et al. 2017; Nikelshparg et al. 2023), the ecomorphological substrate adaptations of the ovipositor are not fully resolved. The most obvious specializations are in the ovipositor length. Wasps probing at greater depths obviously require longer ovipositors (Sivinski and Aluja 2003; Zhen et al. 2005). There is, however, also a need for structural adaptations within the ovipositor to mitigate the forces acting on it during probing; the ovipositor (shaft) must withstand significant mechanical forces and bending moments which vary across substrates. Because the biogenesis of the cuticle is an energetically and resource-intensive process (Wigglesworth 1948; Merzendorfer and Zimoch 2003; Liu et al. 2022; Mullins and Gabbert 2025), selective pressures likely favor a morphology that provide the ovipositor with sufficient durability for a given substrate without incurring unnecessary energetic and resource costs. We therefore expect that the ovipositor morphology reflects an evolutionary trade-off between mechanical robustness and developmental resource economy.

The mechanical properties of the ovipositor can be adapted by either changing its material properties, such as chitin density or the degree of sclerotization, and/or the internal and external geometry. Despite the efficacy of the push–pull mechanism, drilling into tough media is expected to require highly reinforced structures with thicker walls to resist buckling and fracture as well as a large second moment of area (*I*). The latter represents the distribution of the cross-sectional area (CSA) around the neutral axis, which lies in the neutral longitudinal plane where the material undergoes neither tension nor compression during bending. Importantly, this measure captures a strongly nonlinear effect: contributions to *I* increase with the square of the distance from the neutral axis, so material located farther from this axis disproportionately enhances bending stiffness. From a biomechanical perspective, this means that redistributing cuticular material away from the center, rather than simply increasing total mass, is an especially effective strategy to increase resistance to bending and maintain structural integrity during substrate penetration. Likewise, the polar second moment of area quantifies how material is distributed relative to the central axis under torsion, and increases with the squared radial distance of material from this axis, such that placing material farther outward markedly enhances resistance to twisting.

In species drilling in softer materials, these features can be less prominent, and energy and resources can be conserved. Limiting the amount of material in the ovipositor, when possible, also has a beneficial effect on steering capabilities, as it makes it easier to bend the ovipositor. If these adaptive requirements—strong when needed, flexible when possible—are indeed in place, the ovipositor morphology should reflect the substrate the animals probe in. To understand such substrate adaptations, it is essential to study both the ovipositor shaft and the properties of the media they penetrate. While the ovipositor is highly modified at its distal and proximal ends to facilitate penetration and egg passage, the cross-sectional geometry of the shaft varies remarkably little along its length (Eggs et al. 2018, 2023; Cerkvenik, van Leeuwen et al. 2019). Consequently, the flexural rigidity of the shaft can be effectively estimated from a single cross-section.

Here, we investigate to what extent the ovipositor morphology reflects substrate use. Such knowledge elucidates the functional requirements imposed on ovipositors, which in turn makes it possible to generate predictions about the biology of both the parasitoid and its host, and can also facilitate the development of biomimetic applications based on ovipositor design such as (Parittotokkaporn et al. 2009; Scali, Kreeft et al. 2017; Scali, Pusch et al. 2017). Researching substrate adaptations, however, poses several challenges, including the huge diversity of parasitoid wasps (their body size spans across several orders of magnitude), the little-studied host and substrate preferences, the behavioral adaptations of the majority of parasitoid wasps (Compton et al. 2009; Jenner and Roitberg 2009; Barros et al. 2025), and the generally unknown substrate properties. Here, we use 86 previously published ovipositor shaft cross-sections of Ichneumonidae and Braconidae, two of the most species-rich parasitic wasp families (Quicke and van Achterberg 1990; Quicke et al. 1994). We accounted for size-scaling to determine size independent morphology differences that correlate with substrate preference.

## Materials and methods

**Fig. 2:**
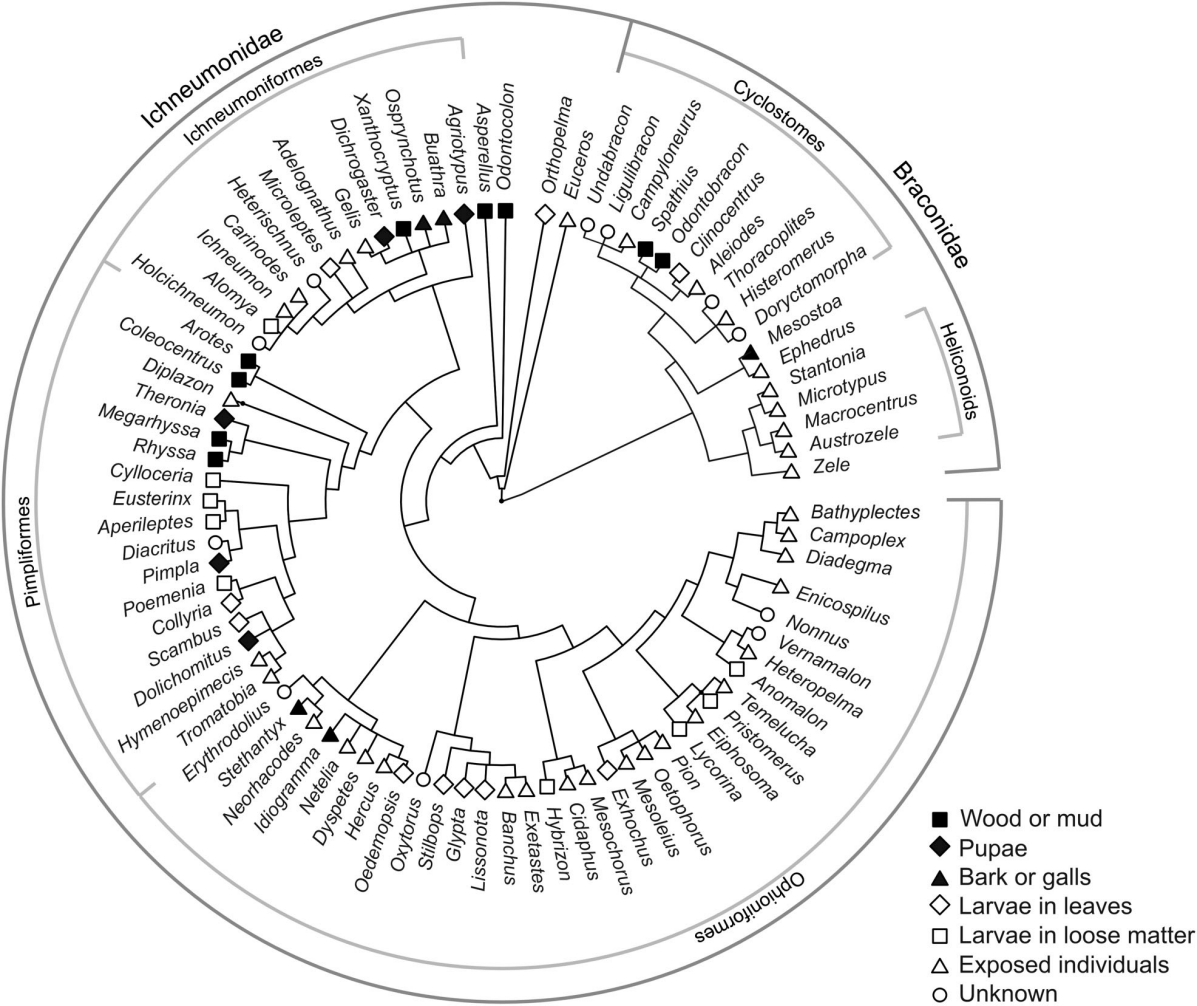
The studied wasp species shown with their phylogenetic relationships and their substrate preferences. The tree was obtained by combining data from (Sharanowski et al. 2011, 2021; Bennett et al. 2019; Zheng et al. 2022) without branch length estimations.

Images of transverse sections of the middle part of the ovipositor, what we will refer to as cross-sections from here on, were obtained from a study describing the hymenopteran ovipositor morphology that included scalebars for size indication (Quicke et al. 1994). A total number of 69 specimens from 35 subfamilies in Ichneumonidae and 17 specimens from 11 subfamilies in Braconidae were used (Fig. 2). For all available species, the preferred host groups and host life histories were obtained from literature (for full data on all species see Table S1). Based on the life histories found, we defined six different classes of probing substrates, based on the location of the host and where the eggs are deposited (Table 1). These oviposition classes vary from penetration in very tough materials such as wood, to a condition in which only the host must be penetrated. This is a coarse and simplified categorization, given the wide variety of substrates in each category. Wood has a toughness (work of fracture) of about 30 kJ/m^2^ which is about 5 times higher than leaves (~6 kJ/m^2^) (Lucas et al. 1991; Pinto Moreira et al. 2017). Values for insect cuticle also vary widely and specific values are hard to find (Vincent and Wegst 2004), but tibia cuticle of locusts has values similar to leaves (~6 kJ/m^2^) (Dirks and Taylor 2012). The work of fracture is probably larger for pupae optimized for defence, and smaller for flexible parts in between tergites and sternites used for penetrating larvae (Lindstedt et al. 2019; Rebora et al. 2023). Therefore our categories show a general decrease in toughness from wood to exposed larvae. Our aim was to create categories which describe the toughest material the wasp has to penetrate. For example, if eggs are laid within a host hidden in wood, we consider the wood the limiting factor. In case a species lays its eggs in an exposed host, the cuticle of the host is the limiting factor. In the case a specific species uses hosts hidden in multiple substrates, we used the toughest of the classes. If the depositing strategy of a species could not be found in literature, it was still included in the analysis but assigned to a seventh class marked as “unknown substrate.”

**Table 1:** Description of substrate classes and the number of species in each group

Substrate class	Description	Toughness	Work of fracture (kJ/m^2^)	No. of species
1	Larvae, deeply concealed in wood or mud nests.	Very tough substrate	~30	10
2	Exposed pupae, chitinous	Tough substrate of animal origin	/	5
3	Larvae, concealed in bark or galls.	Tough substrate of plant origin	/	5
4	Larvae, weakly concealed in leaves	Intermediate substrate of plant origin	~6	12
5	Larvae, weakly concealed in loose soil or decaying matter	Soft substrate	/	7
6	Exposed tissue from larval or adult insects or spiders.	Very soft substrate	<6	36
7	Substrate and life history unknown.	Unknown substrate	/	11

*Note*: Due to the inherent variability of the substrates, the work of fracture values are only indicative of substrate toughness rather than an absolute measure (Lucas et al. 1991; Dirks and Taylor 2012; Pinto Moreira et al. 2017; Rebora et al. 2023).

In the images of the ovipositor cross sections, a total of 22 features were measured or calculated that describe the overall ovipositor morphology (Table 2, Fig. 1). We measured specific features of the olistheter mechanism that links the valves together and those that describe the wall thickness of the valves. Selected parameters related to the olistheter mechanism were the width of the head and neck of the rachis, and the distance between the two rhachides. This distance was scaled to the width of the ovipositor to obtain a better indication of how far to the side or to the middle the olistheter were situated. The position is relevant because olistheters situated on the outside of the ovipositor have larger moment arms compared to those situated close to the center and can therefore withstand larger forces. We also measured the angle of the rachis neck relative to the vertical, with outward pointing rachis given a positive angle, and inward pointing angles a negative value. Both in case of the rachis angle and the parameters for the ventral valves, values of the left and right side were averaged to obtain a single value for further analysis. In addition to several linear measurements, we also determined the CSAs of the exoskeleton and main channels. These were used to calculate the second moment of area over the horizontal and vertical axis (*I_x_* and *I_y_*) of the entire ovipositor and the individual valves as in (Cerkvenik, van Leeuwen et al. 2019). Namely, we used the formula ${I_x} = \mathop \sum \nolimits_i^n {y_i}^2{A_{pix}}$, where *y* is the distance of the pixels from the horizontal axis going through the centroid of the cross-section, *A_pix_* is the area represented by an individual pixel, and *n* is the number of pixels constituting the relevant CSA. The predicted cross-sectional area (CSA_pred_), used as a proxy for ovipositor cross-sectional size, was calculated using the formula for the area of an ellipse ($A = \frac{{\pi \cdot a \cdot b}}{4}$) with *a* being the width and *b* the height of the ovipositor. This was not correlated with the length of the ovipositor but can be used as a proxy to describe structural properties. We used this proxy in our scaling analysis to avoid actual measures that might be affected by the selective drivers we are interested in.

**Table 2:** Description of the parameters and analysis of heterochrony

Char	Description		Expected scaling coefficient	Fitted scaling coefficient	Fitted constant	*R* ^2^ value
	**Overall size parameters (total ovipositor)**					
A	Ovipositor width	Width ovipositor at widest point	0.5	0.476	0.076^a^	0.926
B	Ovipositor height	Height ovipositor at highest point	0.5	0.521	0.029^a^	0.938
CSA_tot_	Cross sectional area	Exo-skeleton and main channels	1	0.988	−0.059^a^	0.976
*I_x_*	Second moment of area (over the central horizontal axis)		2	2.085	−1.483	0.964
*I_y_*	Second moment of area (over the central ventral axis)		2	2.000	−1.367	0.975
	**Olistheter parameters**					
C	Mean rachis head width		0.5	0.527	−1.256	0.742
D	Mean rachis neck width		0.5	0.533	−1.592	0.622
E	Angle of the rachis neck	(not a linear feature so not fitted)				
F	Distance between rhachides	Distance between left and right rachis	0.5	0.426	−0.019^a^	0.630
						
	**Dorsal valve parameters**					
	Cross sectional area (CSA)	Exo-skeleton and main channels	1	1.021	−0.451	0.934
G	Mean lateral wall thickness	Width of the contact area between the dorsal and ventral valves	0.5	0.551	−0.666	0.787
H	Mean dorsal inner wall thickness	Shortest distance from lumen dorsal valve to egg canal	0.5	0.402	−1.066	0.196
I	Mean dorsal outer wall thickness	Shortest distance from lumen dorsal valve to exterior	0.5	0.606	−1.396	0.584
CSA_dv_	Total CSA dorsal channels		1	0.807	−0.263^a^	0.680
*I*_*x*,dv_	Second moment of area (over the central horizontal axis)		2	2.146	−2.518	0.895
*I*_*y*,dv_	Second moment of area (over the central vertical axis)		2	2.082	−1.970	0.947
	**Ventral valve parameters**					
CSA_vv_	Cross sectional area (CSA)	Exoskeleton and main channels	1	0.956	−0.592	0.941
J	Mean ventral inner wall thickness	Shortest distance from lumen ventral valve to egg canal	0.5	0.536	−1.967	0.308
K	Mean ventral outer wall thickness	Shortest distance from lumen ventral valve to exterior	0.5	0.503	−1.224	0.527
CSA_vv,ch_	Mean CSA ventral channels		1	0.769	−0.530	0.637
*I*_*x*,vv_	Second moment of area (over the central horizontal axis)		2	2.00	−2.30	0.924
*I*_*y*,vv_	Second moment of area (over the central vertical axis)		2	1.907	−2.360	0.944

*Note*: Exponential lines were fitted through data of all separate parameters versus the predicted cross-sectional area (Char = *constant* ∙ CSA_pred_^scaling coefficient^). The *constant* indicates the intercept of the fitted line. Expected scaling gives the *scaling factor* expected for an isometric relationship. Result of the line fit are presented as the fitted scaling coefficient, fitted constant, and the *R*^2^ value of the fit.

All morphological features, except height, width, and area, were measured using Olympus AnalySIS^D 2.2 build 1089 (Olympus Corp., Waltham, USA). The width, height, and area parameters were determined with a custom algorithm in MATLAB R2023b (MathWorks, Natick, MA, USA). Statistical analysis was done in R-studio (Posit team, version 2025.09.1) running R version 4.4.2.

To test for the effect of substrate on each ovipositor character, we performed phylogenetic generalized least squares (PGLS) analyses. For each character, we fitted a model (character ~ substrate) using maximum likelihood (ML) estimation of Pagel’s λ, which quantifies the strength of phylogenetic signal in the residuals (Pagel 1999). Analyses were conducted using the *pgls* function (package *caper*), which performs PGLS regression. The phylogenetic tree for this analysis was assembled by combining the topological relationships reported in previously published molecular phylogenies (Sharanowski et al. 2011, 2021; Bennett et al. 2019; Zheng et al. 2022); see Fig. 2 and SI. No independent phylogenetic analysis was done because independent data was not available for all species in this study. Consequently, the resulting tree is a topology-only synthesis in which all branch lengths were set to 1. Therefore, the analyses account for phylogenetic relatedness among taxa based on tree topology, but do not incorporate information on divergence times or evolutionary distances.

When the PGLS model indicated a statistically significant effect of Substrate (*P* < 0.05), we conducted post hoc pairwise comparisons. For these analyses, we used the *gls* function (package *nlme*) to fit an equivalent generalized least squares (GLS) model incorporating a Pagel’s λ correlation structure fixed at the ML estimate obtained from the PGLS model. This approach ensured consistency in the phylogenetic correction between the global test and subsequent comparisons.

Post hoc differences among substrate levels were evaluated using Tukey’s honestly significant difference tests implemented via general linear hypotheses, using the *glht* function (package *multcomp*). To test for heterochrony, we analyzed the relation between the predicted cross-sectional area (CSA_pred_), the proxy for overall size, and the other parameters. For all parameters, we fitted an exponential curve (*y* = *c* ∙ CSA_pred_^*k*^, with *c* being the constant and *k* the scaling coefficient/growth factor) and compared the growth factor to the factors we expected based on isometric growth. Residuals of the fits were saved and used for further comparisons between substrate groups to eliminate size effects.

## Results

### Overall size and heterochrony

**Fig. 3:**
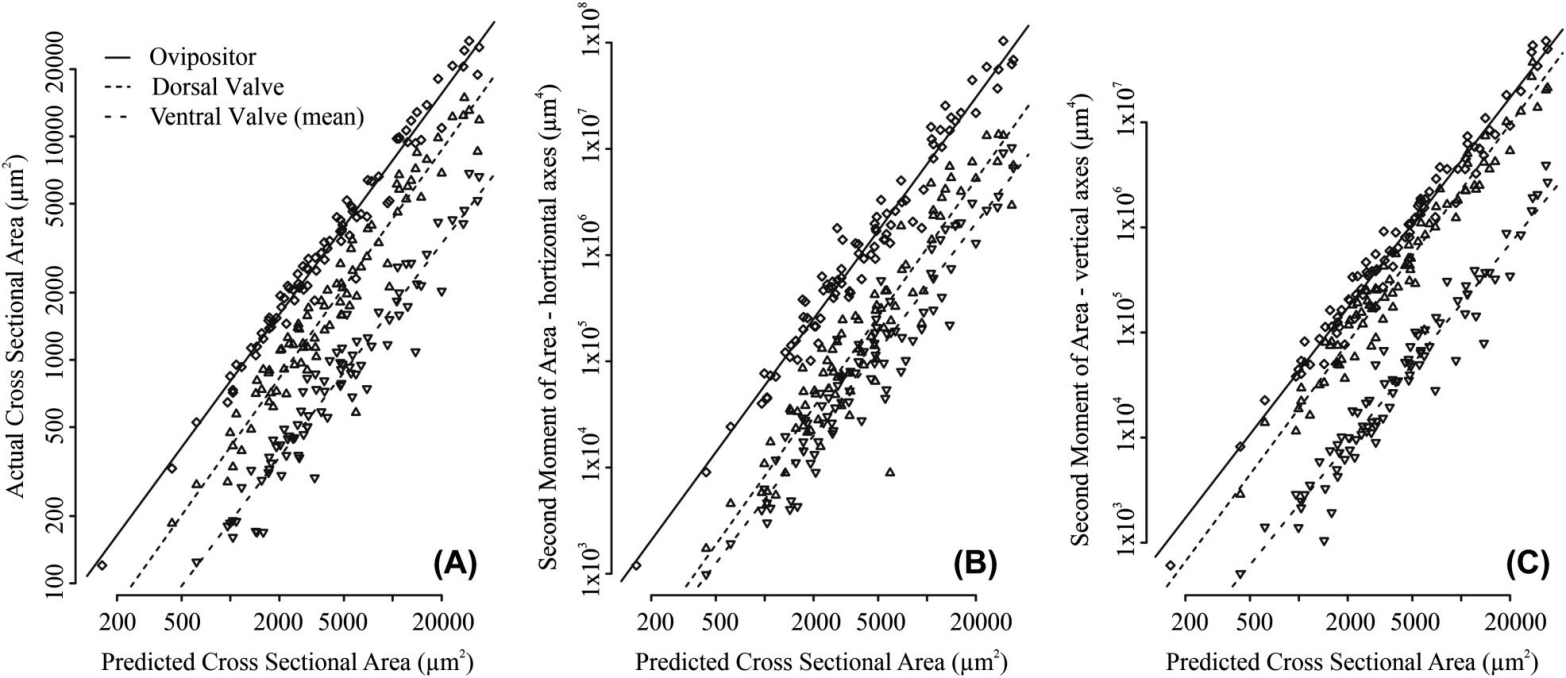
Scaling of the ovipositor, the dorsal, and mean ventral valve to the theoretical CSA estimated from the ovipositor width and height. (A) Scaling of the CSA consisting of exoskeleton and main channels. Scaling factors—Isometry: 1, Total ovipositor: 0.99, Dorsal Valve: 1.02, Ventral Valve: 0.96. (B) Scaling of the structural stiffness indicator second moment of area over the central horizontal bending axis (*I_x_*). Scaling factors—Isometry: 2, Total ovipositor: 2.08, Dorsal Valve: 2.15, Ventral Valve: 2.00. (C) Scaling of the structural stiffness indicator second moment of area over the vertical horizontal bending axis (*I_y_*) Scaling factors—Isometry: 2, Total ovipositor: 2.00, Dorsal Valve: 2.08, Ventral Valve: 1.91.

Almost all parameters scale isometric with CSA_pred_ of the ovipositor, indicating no shape differences between large and small ovipositors (Table 2). Both the actual CSA and the structural strength—determined by second moment of area—of the different valves do not change with a change in size of the predicted CSA (Fig. 3). The sizes of both the dorsal and ventral channels show slight negative allometry, while the dorsal outer wall thickness shows positive allometry, indicating that, as body size increases, the channels become relatively smaller whereas the outer walls become relatively thicker.

**Fig. 4:**
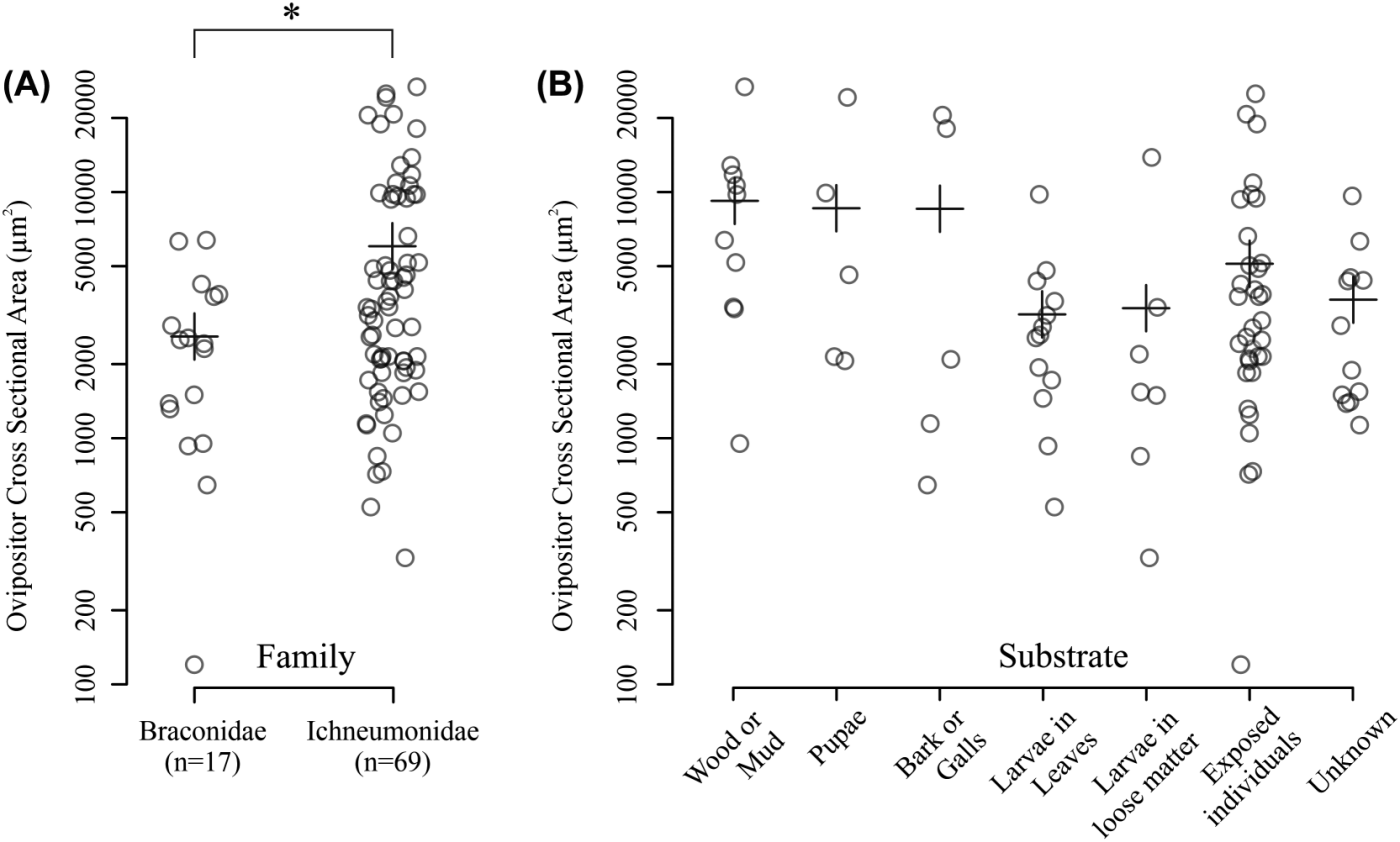
Ovipositor size (as CSA) comparison between families (left) and substrate preferences (right). Significant differences were found between the two families (*P* < 0.05), while no significant size differences were found between animals with different substrate preferences.

There was a difference in size between Braconidae and Ichneumonidae (Fig. 4A, *t*-test, *t* = −2.488, *df* = 84, *P* = 0.015), probably due to the lower overall number of investigated braconid species and lower number of large braconid species. We tested for differences in ovipositor cross–sectional area among substrate types using phylogenetic PGLS. Substrate had no significant effect on ovipositor size after phylogenetic correction (Fig. 4B, PGLS: *df* = 6, *F* = 1.50, *P* = 0.19), indicating that size is not a good indicator for substrate penetration preference. In contrast, ovipositor cross-sectional size showed a moderate phylogenetic signal (λ = 0.61, 95% CI = 0.01–0.94), indicating that size might be taxon specific.

### Olistheter adaptations to substrates

**Fig. 5:**
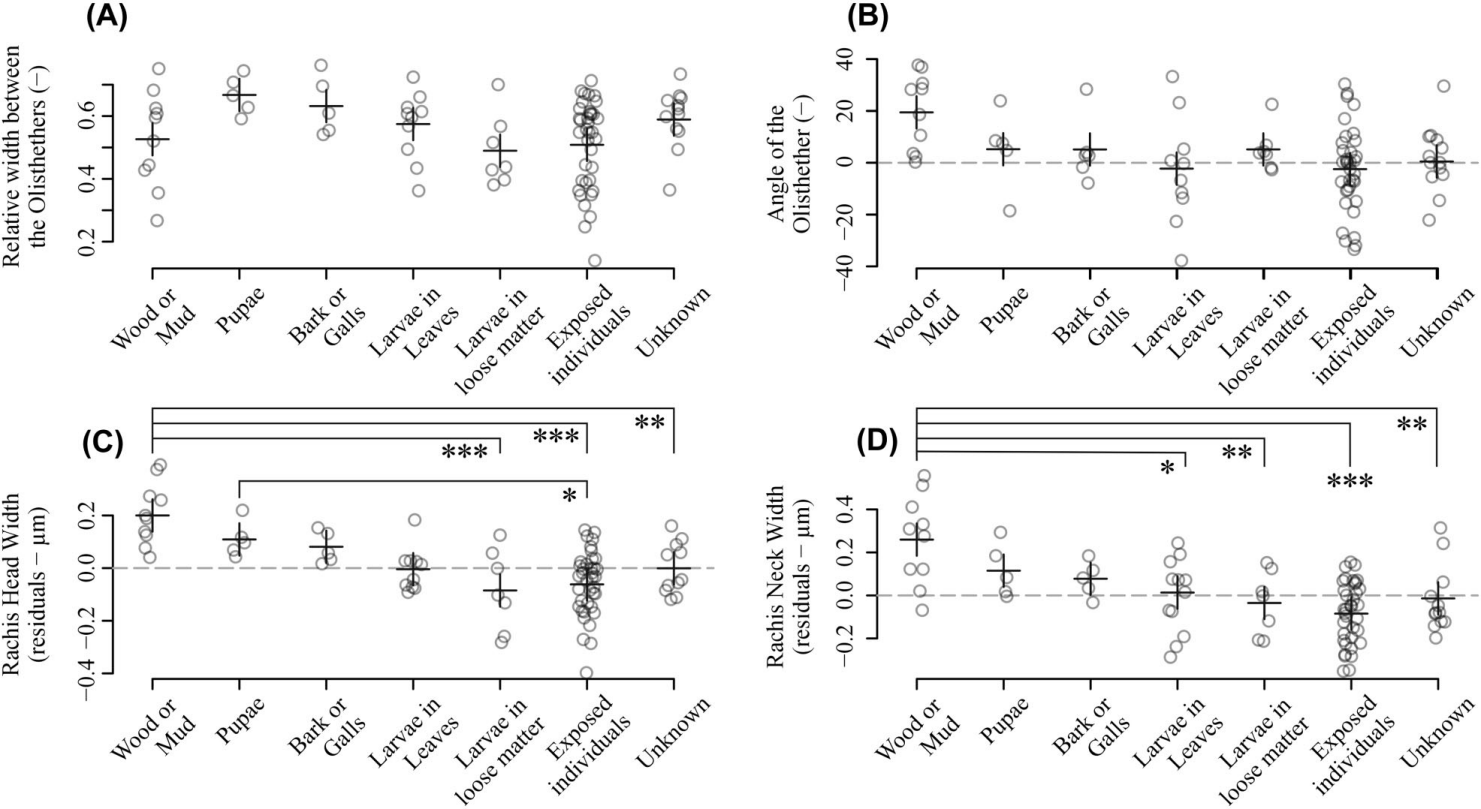
Comparison of the olistheter parameters between groups with different substrate preferences. Comparison of the olistheter parameters between groups with different substrate preferences. Circles indicate species, the crosses indicate the means. * = *P* < 0.05, ** = *P* < 0.01, *** = *P* < 0.001. (A) Relative width between the left and right olistheter, no significant differences were found. (B) Mean angle of the rhachis neck, with positive angles indicating outward pointing rhachides. The dashed line indicates vertical orientation. (C, D) Rhachis head and neck widths (residual values of the scaling line fit). The dashed line at residual zero indicates the fitted value. The rhachides of the species probing in the toughest substrate were significantly wider than those from the three (C) or two (D) groups with the lowest values.

In further analyses, we use the residuals of the line-fit to exclude any size effects. Henceforth, whenever referring to a parameter we imply the residuals, unless the data was not size dependent such as in ratios and angles. A comparison of the olistheter parameters across substrates showed significant differences in two out of the four investigated features (PGLS results in Table 3). Post-hoc testing (Tukey-adjusted contrasts based on a GLS model incorporating the estimated phylogenetic correlation structure) for the parameters showing significant differences between substrates showed that animals using the very tough substrates differed from other groups (Table 3, Fig. 5). In “wood and mud” probing animals, we observed wider rachis heads and necks. Wood–probing species also seem to have more outwardly pointing rachides although this pattern was not statistically supported (Fig. 5B). Although no other significant differences were found, the distribution of the data points suggests a potential correlation: animals probing in tougher substrates tend to exhibit thicker and more outwardly oriented rachides heads and necks.

**Table 3:** Differences in morphology related to preferred substrate

	PGLS results				Post-hoc (Tukey) results	
Description	*df*	*F*	*P*	λ	Group comparison	*P*
**Olistheter chracteristics**						
Mean rachis head width	6	7.57	<0.001	0.62	Wood or mud vs. Larvae in loose matter Wood or mud vs. Exposed individuals Wood or mud vs. Unknown substrate Pupae vs. Exposed individuals	<0.001 <0.001 0.003 0.049
Mean rachis neck width	6	4.89	<0.001	0.47	Wood or mud vs. Larvae in Leaves Wood or mud vs. Larvae in loose matter Wood or mud vs. Exposed individuals Wood or mud vs. Unknown substrate	0.031 0.008 <0.001 0.007
Angle of the rachis neck	6	1.70	0.133	0.80		
Relative distance between rhachides	6	2.00	0.076	0.03		
**Dorsal valve parameters**						
Mean lateral wall thickness	6	3.44	0.005	0.88	(−)	
Mean dorsal inner wall thickness	6	0.55	0.766	0.52		
Mean dorsal outer wall thickness	6	3.56	0.004	0.73	Wood or mud vs. Larvae in loose matter	0.016
Second moment of area (over the central horizontal axis, *I*_*x*,dv_)	6	2.30	0.041	0.23	(−)	
Second moment of area (over the central vertical axis, *I*_*y*,dv_)	6	5.13	<0.001	1.00	Pupae vs. Larvae in loose matter Pupae vs. Exposed individuals Bark and Galls vs. Exposed individuals	0.027 0.003 0.018
**Ventral valve parameters**						
Mean ventral inner wall thickness	6	1.06	0.393	0.43		
Mean ventral outer wall thickness	6	1.19	0.319	0.88		
Second moment of area (over the central horizontal axis, *I*_*x*,vv_)	6	0.76	0.601	0.79		
Second moment of area (over the central vertical axis, *I*_*y*,cv_)	6	1.05	0.398	0.63		

*Note*: Group differences were analyzed using PGLS, accounting for shared ancestry among taxa. Pairwise comparisons among substrate categories were conducted using Tukey-adjusted contrasts based on a GLS model incorporating the estimated phylogenetic correlation structure. For the pairwise comparisons, only the pairs that are significantly different are reported.

### Adaptations in valve morphology

As mentioned above, there is an isometric size relationship in both overall CSA and second moment of area. As all individual valves are alternatively pushed into the substrate, one might expect adaptations in the valves, especially when they are used to penetrate tougher substrates. However, we found no differences in ventral valve features between animals probing different substrates (PGLS, results Table 3). The dorsal valve, however, does show significant morphological differences between animals using different substrates (PGLS, results Table 3), specifically in the outer wall of the ovipositor which is described by the lateral wall parameter and the width of the dorsal wall (PGLS, results Table 3). The differences are small, because in post-hoc testing (Tukey-adjusted contrasts based on a GLS model incorporating the estimated phylogenetic correlation structure) no significant differences were found in the mean lateral wall thickness. The significant difference we found for the dorsal wall thickness show that the wall is thicker in animals that probe in wood and mud compared to animals probing in larvae hiding in loose material.

**Fig. 6:**
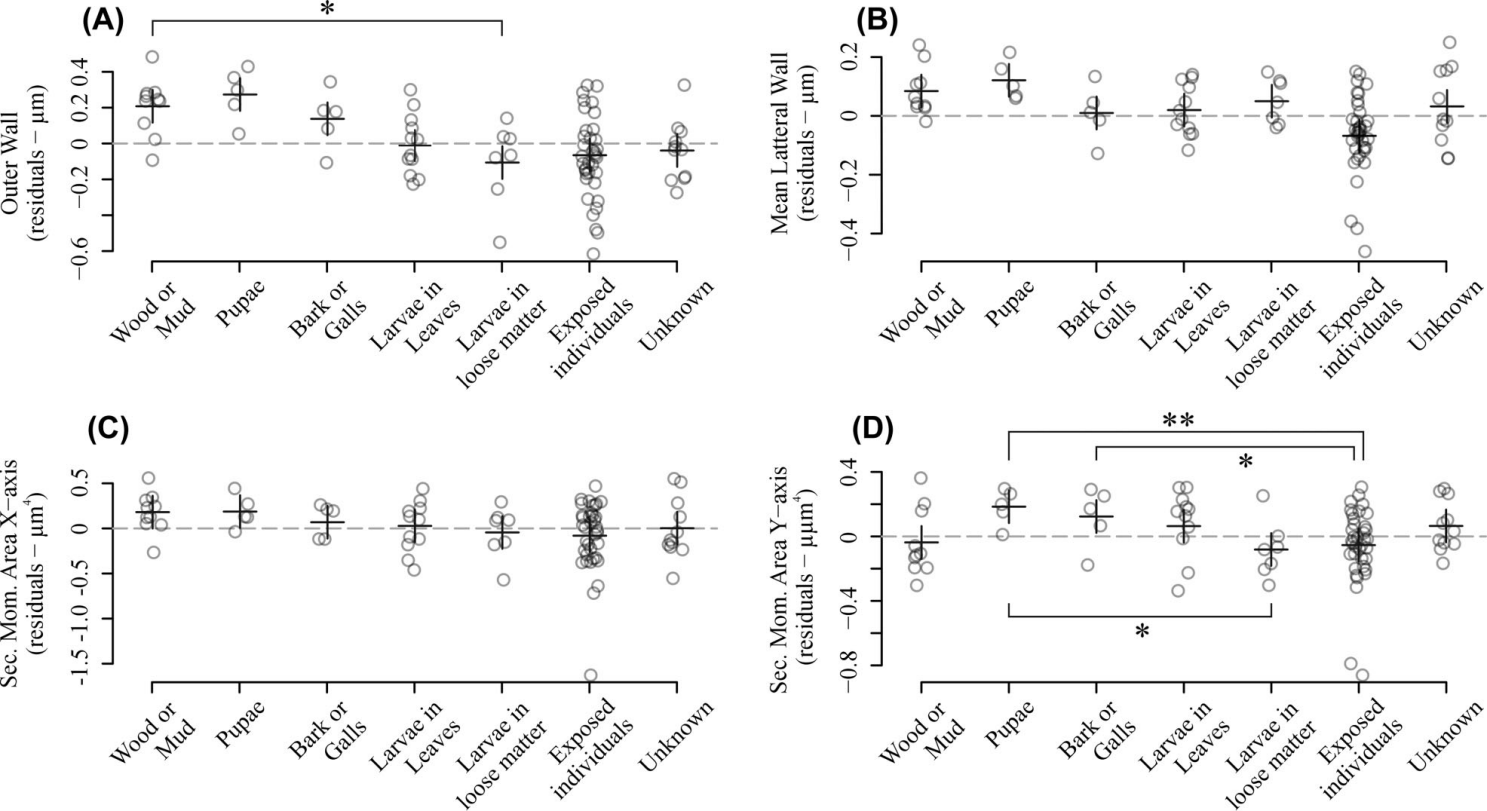
Comparison of the dorsal valve features between groups with different substrate preferences. Comparison of the dorsal valve parameters between groups with different substrate preferences. Circles indicate species, the crosses indicate the mean values. Plotted are the residuals of the scaling line fit. The dashed line at residual zero indicates the fitted value. (A) Outer wall thickness of the species probing in pupae was significantly thicker than those of species probing in larvae hidden in loose matter (* = *P* < 0.05). (B) Inner wall thickness. No significant differences between groups were found. (C) Second moment of area in *x* shows no significant differences across substrates. (D) Second moment of area in *y* shows significant differences between species probing in exposed hosts compared to species that probe either the pupae (** = *P* < 0.01) or bark/galls (* = *P* < 0.05). Species probing in pupae also differ when compared to those probing for larvae in loose matter (* = *P* < 0.05).

The thicker dorsal wall seems to correlate with a higher second moment of area especially over the *Y* axis where a significant difference was found between animals probing in pupae and in exposed individuals (Table 3, Fig. 5D). Generally, there is a tendency for thicker outer walls and higher structural stiffness with an increase in substrate toughness (Fig. 6, Table 3).

## Discussion

Understanding how the environment shapes organismal form and function is what evolutionary biologists strive for. Our morphometric analysis of ovipositors from 86 parasitic hymenopteran species and linking them to probing substrate characteristics, provides a first indication of selective drivers that lead to specific adaptations in the morphological (and structural) properties of the ovipositor. The morphological differences were most prominent in the olistheter, and to a smaller extent in the wall thickness of the dorsal valve, when comparing substrate extremes.

The conserved gross cross-sectional anatomy across a large size range suggests that the push–pull mechanism is an optimized solution for substrate puncturing and exploration independent of size (Fig. 3). Nevertheless, the observed negative allometry in the size of the internal channels (Table 2) indicates that larger ovipositors possess relatively thicker outer walls, but with a very small effect on the size corrected second moment of area (*I_x_* and *I_y_*). This could be because chitin deposition is metabolically expensive (Wigglesworth 1948; Merzendorfer and Zimoch 2003; Liu et al. 2022; Mullins and Gabbert 2025). Therefore, simply scaling up every part of the ovipositor valve is a rather inefficient use of resources. The expansion of the channel toward the inner wall, instead toward the outer wall, might be a strategy of effective resource allocation. These channels are essential for metabolic and sensory functions, housing the trachea and nerves transmitting information detected by sensilla (Larocca et al. 2007; Dweck et al. 2008; Ahmed et al. 2013; Alves et al. 2014; Yadav and Borges 2017). By placing the thinnest part of the valve closest to the neutral (bending) axis—where material contributes the least to the shaft’s bending resistance—the functional channel volume is preserved with negligible impact on structural stiffness. Furthermore, placing the nerves close to the neutral axis also ensures they are minimally affected by the potential bending of the ovipositor. This allows the ovipositor to maintain its sensory function while prioritizing the maximum mechanical reinforcement.

Our results show a significant difference in CSAs between Braconidae and Ichneumonidae (Fig. 4A). However, due to the large difference in group sizes, we cannot conclude that this represents a true phylogenetic effect, but we see a high λ-score in the PGLS confirming that size is indeed phylogenetically determined. Also, in the analysis of the other parameters, we find high λ-scores indicating large phylogenetic effects (Table 3). It would be interesting to explore whether such effects are present across all parasitic Hymenoptera by employing more comprehensive phylogenetic coverage in future studies. While absolute ovipositor size is not an indicator for substrate preference (Fig. 4B), three olistheter parameters showed clear substrate specializations (Fig. 5). Specifically, wider heads and necks of rhachides as well as a more lateral position were indicative for animals probing in tougher substrates. This reinforcement likely prevents valve separation during probing. Puncturing and drilling into tough substrates requires high axial loads which, when resisted, generate lateral forces that could separate the tongue-and-groove joint. The robust, laterally positioned rhachides appear specifically optimized to counteract this deflection, maintaining olistheter integrity during probing in tougher substrates.

The thickness of the dorsal wall, expressed as lateral wall thickness, and dorsal outer wall thickness showed a similar pattern to the olistheter parameters. Here, it also seems that animals probing in tough substrates have a more robust morphology (Fig. 6), although for some parameters this is not true for animals probing in wood and mud. This could be due to the high variability in such materials. Admittedly, we considerably simplified substrate classifications—organic materials are complex, nonhomogeneous, and their material properties can vary in time. For example, wasps probing in fresh wood experience drastically different mechanical resistance compared to those probing in decaying wood. Similarly, the mineral and water content of mud likely affects its toughness. A more detailed substrate classification, supported by toughness measurements, could uncover additional statistically significant morphological adaptations. Good candidates are the orientation and relative position of the olistheter mechanism within the ovipositor, which currently approached statistical significance across substrates (Figs. 5 and 6).

Thick chitinous cuticles encapsulate and protect many holometabolous insects during metamorphosis, an important process during which insects are highly vulnerable. In fact, the pupal cuticle can be made resistant to puncture by making it smooth, heavily sclerotized, and even mineralized (Vincent and Wegst 2004; Clark and Triblehorn 2014; Rong et al. 2019). For parasitic wasps that exploit such pupal hosts, the dorsal valve likely functions primarily as a puncturing device, with only limited need for steering. In these instances, the dorsal valve may act as the primary structural stabilizer against the bending moments encountered during puncturing.

The reinforcements we observed in the rhachides and dorsal walls create a trade-off between substrate penetration and ovipositor maneuverability. While some species may rely on straight-line drilling, maneuverability is a critical requirement for wasps that must navigate winding paths through complex substrates (Compton and Nefdt 1988; Kundanati and Gundiah 2014; Cerkvenik et al. 2017), or precisely target specific regions, such as the brain ganglia in the host (Haspel et al. 2003). These tasks require significant ovipositor flexibility; however, the structural stiffening needed to withstand high axial loads inevitably constrains steering capability. This may explain why the “wood and mud” penetrating species have smaller rigidity and more medially placed olistheters than wasp species that exploit pupal hosts. The former need to steer their way through a substrate, while for the latter a straight insertion is sufficient.

Although several steering mechanisms have been proposed, their *in vivo* efficacy remains largely untested (Quicke 1991; Quicke and Marsh Paul 1992; Quicke and Fitton 1995; Quicke et al. 1995; Cerkvenik, Dodou et al. 2019). For at least one species, it has been shown that steering is primarily controlled by adjusting the valve offset, where the interplay between insertion forces and substrate reaction generates bending moments at the tip (Cerkvenik et al. 2017; Cerkvenik, van Leeuwen et al. 2019). We predict that the species identified as having the most robust cross-sections are likely the most limited in their maneuverability.

Interestingly, we found no significant differences in ventral valve features across substrate groups. This lack of variation could indicate the valves’ distinct functional roles during drilling. Specifically, the dorsal valve may serve as the primary structural stabilizer under compression, while the ventral valves provide support primarily under tension, as chitin fibers in the ovipositor are oriented along its longitudinal axis (Blackwell and Weih 1980). Longitudinal fibers are well suited for mitigating tensile strength. The increased wall thickness of the dorsal valve appears to be a structural adaptation providing the necessary flexural rigidity to resist compressive loads during probing in tough substrates. In contrast, the ventral valves with their serrated tips (Vincent and King 1995; Ghara et al. 2011; Eggs et al. 2018; Cerkvenik, Dodou et al. 2019) likely rely on the high tensile strength and therefore remain morphologically consistent across different media.

Furthermore, the configuration of the internal ovipositor channels is likely influenced by the egg deposition function. Parasitic wasp eggs can be large and need to be deformed to pass through a narrow egg channel (Fulton 1933; King et al. 1968; Nikelshparg et al. 2023). This could result in significant internal pressure on the valves and the olistheter. Having thinner inner walls, which have been shown to contain resilin (Cerkvenik, van Leeuwen et al. 2019), might serve a dual purpose. In addition to considerations of material allocation, compliant inner walls may deform during egg passage, redistributing contact forces and dissipating mechanical energy, thereby reducing peak stresses on the olistheter.

In conclusion, several extrinsic factors influence ovipositor morphology that can be explained by the mechanical loading of the ovipositor while reaching their hosts to successfully deposit an egg. Penetrating though substrates is correlated with a sturdy olistheter mechanism and relatively thick walls in the dorsal valve. However, these factors may limit steering possibilities. Species with the most robust dorsal valves and olistheters penetrate high-resistance media, such as pupal cuticles, probably without the need for steering. If the latter is necessary—for example, in species probing in wood and mud—some of the ovipositor structural safety is jeopardized to accommodate this need. Selective pressures on the ventral valves may be relatively limited for the parameters examined here, given the weak morphological sensitivity to their variation. Instead, adaptation to longitudinal tensile stresses and the requirements of egg passage might be the primary factors shaping these structures. Ultimately, the anatomy of the ovipositor reflects a set of functional trade-offs that tune its flexural rigidity to balance the competing requirements of maneuverability, substrate penetration, and the mechanical constraints associated with egg transport. Using this knowledge, we can better understand and predict the life-history strategies of Hymenoptera. Additionally, insights into substrate-specific adaptations can inform the design of improved steerable needles for medical applications.

## Supplementary Material

icag129_Supplemental_File

## Data Availability

All data supporting the findings of this study are available within the paper, references, and its Supplementary Information.

## Notes

From the symposium “Needle in the haystack: Finding tools to study puncture and injection mechanisms across biology” presented at the annual meeting of the Society for Integrative and Comparative Biology, January 3–7th, 2026.
