# Supplementary material for: Slender but Strong: Substrate-Driven Adaptations in Parasitic Wasp Ovipositors
Source: Integr Comp Biol. 2026 Jul 23;66:icag129. doi: 10.1093/icb/icag129 (PMC13452084; doi:10.1093/icb/icag129)
Supplement: Supplementary file 1 [file icag129_supplemental_file.docx]

**Supplementary material**

**SI Table 1.** Investigated species, their taxonomic classification, and biological information. In the column »Family«, braconids are denoted with B, ichneumonids with I.

| **Family** | **Higher group** | **Subfamily** | ***Genus*** | **Life history strategy** | **Section source** | **Host group** | **Substrate group** | **Additional info** | **Host source** |
| --- | --- | --- | --- | --- | --- | --- | --- | --- | --- |
| B | Aphidoid complex | Aphidiinae | *Ephedrus* (Haliday) | Endoparasitoid | 1,2 | Hemiptera: Aphididae | Exposed individuals |  | (Dureseau and Rivet 1972; Chow and Mackauer 1986) |
| B | Aphidoid complex | Mesostoinae | *Mesostoa* (Van Achterberg) | Ectoparasitoid | 1 | Phytophagous, gall wasp | Bark or Galls | Young woody plant shoots | (Dangerfield and Austin 1998) |
| B | Cyclostomes | Braconinae | *Campyloneurus* (Szepligeti) | Ectoparasitoid | 1 | Lepidoptera: Crambidae | Exposed individuals | Nymphs/adults in waxy scales | (Saxena 1967) |
| B | Cyclostomes | Braconinae | *Ligulibracon* (Quicke) | Unknown | 1 | Unknown | Unknown |  |  |
| B | Cyclostomes | Braconinae | *Undabracon* (Quicke) | Unknown | 1 | Unknown | Unknown |  |  |
| B | Cyclostomes | Doryctinae | *Odontobracon* (Quicke) | Idiobiont ectoparasitoid | 1 | Probably Coleoptera | Wood or Mud | Probably larvae | (Marsh 1970) |
| B | Cyclostomes | Doryctinae | *Spathius* (Nees) | Idiobiont ectoparasitoid | 1 | Coleoptera: Buprestidae | Wood or Mud | Larvae | (Wang et al. 2010; Yang et al. 2010) |
| B | Cyclostomes | Mesostoinae | *Doryctomorpha* (Ashmead) | Unknown | 1 | Unknown | Unknown |  |  |
| B | Cyclostomes | Rhyssalinae | *Thoracoplites* (Fischer) | Unknown | 1 | Unknown | Unknown |  |  |
| B | Cyclostomes | Rhyssalinae | *Histeromerus* (Wesmael) | Idiobiont (?) ectoparasitoid | 1 | Coleoptera | Exposed individuals | Larvae | (Shaw 1995b) |
| B | Cyclostomes | Rogadinae | *Aleiodes* | Koinobiont endoparasitoid | 1 | Lepidoptera: Geometridae | Exposed individuals | Larvae | (Fortier 2006, 2008) |
| B | Cyclostomes | Rogadinae | *Clinocentrus* (Haliday) | Endoparasitoid | 1 | Lepidoptera: Epermeniidae | Larvae in leaves | Larvae | (Shaw 1981; Shaw and Aeschlimann 1994) |
| B | Euphoroid complex | Euphorinae | *Zele* (Curtis) | Koinobiont endoparasitoid | 1,2 | Lepidoptera: Noctuidae | Exposed individuals | Larvae | (van Achterberg 1979) |
| B | Helconoid complex, Macrocentroid subcomplex | Macrocentrinae | *Austrozele* (Roman) | */* | 1 | Lepidoptera: Noctuidae | Exposed individuals |  | (van Achterberg 1993; Barbosa et al. 2001; Barbosa and Caldas 2004) |
| B | Helconoid complex, Macrocentroid subcomplex | Macrocentrinae | *Macrocentrus* (Curtis) | Koinobiont endoparasitoid | 1,2 | Lepidoptera: Crambidae | Exposed individuals | Larvae | (Cossentine and Lewis 1987; van Achterberg 1987; Ding et al. 1989) |
| B | Helconoid complex, Macrocentroid subcomplex | Microtypinae | *Microtypus* (Ratzeburg) | Koinobiont endoparasitoid | 1 | Lepidoptera: Pyralidae | Exposed individuals | Larvae | (Čapek and van Achterberg 1992; Murray et al. 2017) |
| B | Helconoid complex, Macrocentroid subcomplex | Orgilinae | *Stantonia* (Ashmead) | Koinobiont endoparasitoid | 1 | Lepidoptera: Crambidae | Exposed individuals | Larvae | (Kula et al. 2010) |
| I | Ichneumoniformes | Adelognathinae | *Adelognathus* (Holmgren) | Konobiont/ Idiobiont ectoparasitoid | 1 | Hymenoptera: Thentredenidae | Exposed individuals | Larvae | (Rahoo and Luff 1987) |
| I | Ichneumoniformes | Agriotypinae | *Agriotypus* (Curtis) | Idiobiont ectoparasitoid | 1 | Trichoptera: Goeridae | Pupae |  | (Elliott 1982) |
| I | Ichneumoniformes | Cryptinae | *Buathra* (Cameron) | Idiobiont ectoparasitoid | 1 | Hymenoptera: Sphecidae | Mud | Eggs in soil nests | (Casiraghi et al. 2001) |
| I | Ichneumoniformes | Cryptinae | *Dichrogaster* (Doumerc) | Idiobiont ectoparasitoid | 1 | Neuroptera: Chrysopidae, Hemerobiidae | Pupae | Cocoons | (Panteloni 1987) |
| I | Ichneumoniformes | Cryptinae | *Gelis* (Thunberg) | Idiobiont ectoparasitoid | 1 | Parasitoid Araneae or hyperparasitoid on Hymenoptera in wide range of hosts | Exposed individuals | Egg sacs/Cocoons | (Weseloh 1986; van Baarlen et al. 1996) |
| I | Ichneumoniformes | Cryptinae | *Osprynchotus* (Mocsary) | Idiobiont ectoparasitoid | 1 | Hymenoptera: Aculeata | Bark or Galls | Larvae | (Ghigi 1911; Santos and Aguiar 2013; Wahl and Green 2020) |
| I | Ichneumoniformes | Cryptinae | *Xanthocryptus* (Cameron) | Idiobiont | 1 | Coleoptera: Cerambycidae | Wood or Mud | Larvae | (Schreurs 1965; Valentine 1967; Wang and Shi 2001) |
| I | Ichneumoniformes | Ichneumoninae | *Alomya* (Panzer) | Endoparasitoid | 1 | Lepidoptera: Hepialidae | Larvae in loose matter |  | (Cameron 1950; Hinz and Short 1983) |
|  | Ichneumoniformes | Ichneumoninae | *Carinodes* (Hancock) | Endoparasitoid | 1 | Lepidoptera: Noctuidae | Exposed individuals | Larvae | (Harrison 1963) |
| I | Ichneumoniformes | Ichneumoninae | *Heterischnus thoracicus* (Gravenhorst) | Unknown | 1 | Unknown | Unknown |  |  |
| I | Ichneumoniformes | Ichneumoninae | *Holcichneumon areolator* (Heinrich) | Unknown | 1 | Unknown | Unknown |  |  |
| I | Ichneumoniformes | Ichneumoninae | *Ichneumon* (Ashmead) | Idiobiont endoparasitoid | 1 | Lepidoptera: Lycaenidae | Exposed individuals | Larvae | (Thomas and Elmes 1993) |
| I | Labeniformes | Labeninae | *Asperellus* (Townes) | Idiobiont, ectoparasitoid | 1 | Coleoptera | Wood or Mud | Larvae | (Gauld and Wahl 2000) |
| I | Ophioniformes | Anomaloninae | *Anomalon* (Panzer) | Koinobiont endoparasitoid | 1 | Lepidoptera: Tortricidae or/and Coleoptera | Larvae in loose matter |  | (Sonan 1930; Gauld 1978, 1980; Quicke 2014) |
| I | Ophioniformes | Anomaloninae | *Heteropelma* (Wesmael) | Koinobiont endoparasitoid | 1 | Lepidoptera: Noctuidae | Exposed individuals | Larvae | (Johns and Whitehouse 2004) |
| I | Ophioniformes | Anomaloninae | *Vernamalon* (Gauld) | Unknown | 1 | Unknown | Unknown |  |  |
| I | Ophioniformes | Banchinae | *Banchus* (Fabricius) | Koinobiont endoparasitoid | 1 | Lepidoptera: Noctuidae | Exposed individuals | Larvae | (Arthur and Ewen 1975; Arthur and Mason 1985) |
| I | Ophioniformes | Banchinae | *Exetastes* (Gravenhorst) | Koinobiont endoparasitoid | 1 | Lepidoptera: Noctuidae | Exposed individuals | Larvae | (Buleza 1990) |
| I | Ophioniformes | Banchinae | *Glypta* (Gravenhorst) | Koinobiont endoparasitoid | 1 | Lepidoptera: Tortricidae | Larvae in leaves |  | (McGugan 1955; Miller 1960; Stairs 1983) |
| I | Ophioniformes | Banchinae | *Lissonota* (Gravenhorst) | Koinobiont endoparasitoid | 1 | Lepidoptera | Larvae in leaves |  | (Zong et al. 2012) |
| I | Ophioniformes | Campopleginae | *Bathyplectes* (Thomson) | Koinobiont endoparasitoid | 1 | Coleoptera: Curcullionidae | Exposed individuals | Larvae | (Putiler 1974; Berberet 1982) |
| I | Ophioniformes | Campopleginae | *Campoplex* (Gravenhorst) | Koinobiont endoparasitoid | 1 | Lepidoptera: Gelechiidae | Exposed individuals | Larvae | (Leong and Oatman 1968; Wang and Shi 2001) |
| I | Ophioniformes | Campopleginae | *Diadegma* (Foerster) | Koinobiont endoparasitoid | 1 | Lepidoptera: Plutellidae | Exposed individuals | Larvae | (Idris and Grafius 1996; Dancau et al. 2020) |
| I | Ophioniformes | Cremastinae | *Eiphosoma* (Cresson) | Koinobiont endoparasitoid | 1 | Lepidoptera: Noctuidae | Exposed individuals | Larvae | (Giraldo-Vanegas and García R 1994; Allen et al. 2021) |
| I | Ophioniformes | Cremastinae | *Pristomerus* (Curtis) | Koinobiont endoparasitoid | 1 | Lepidoptera: Tortricidae, Gelechiidae | Larvae in leaves |  | (Polaszek et al. 1994; Rousse et al. 2013, 2013) |
| I | Ophioniformes | Cremastinae | *Temelucha* (Foerster) | Koinobiont endoparasitoid | 1 | Lepidoptera: Olethreutidae, Gelechiidae | Exposed individuals | Sometimes exploits wounds made by other parasitoids | (Oatman and Platner 1974; Ashley et al. 1982) |
| I | Ophioniformes | Ctenopelmatinae | *Mesoleius* (Holmgren) | Koinobiont endoparasitoid | 1 | Hymenoptera: Tenthredinidae | Exposed individuals | Larvae | (Criddle 1928; Muldrew 1953) |
| I | Ophioniformes | Ctenopelmatinae | *Oetophorus* (Foerster) | Koinobiont endoparasitoid | 1 | Hymenoptera: Tenthredinidae | Exposed individuals | Larvae | (Barron 1997) |
| I | Ophioniformes | Ctenopelmatinae | *Pion* (Schiødte) | Koinobiont endoparasitoid | 1 | Symphyta: Tenthredinidae | Exposed individuals | Eggs/Larvae | (Hinz 1961; Quicke 2014) |
| I | Ophioniformes | Hybrizontinae | *Hybrizon* (Fallén) | Endoparasitoid | 1 | Hymenoptera: Formicidae | Larvae in loose matter |  | (van Achterberg and Durán 2011) |
| I | Ophioniformes | Lycorininae | *Lycorina* (Holmgren) | Koinobiont endoparasitoid | 1 | Lepidoptera: Tortricidae | Larvae in leaves |  | (Chao 1980) |
| I | Ophioniformes | Mesochorinae | *Cidaphus* (Foerster) | Koinobiont endoparasitoid (obligate hyperparasitism) | 1 | Hyperparasitoid of Hymenoptera, I (in Lepidoptera, Caradrinidae) | Exposed individuals | Hosts that are (pre)parasitized by other ichneumonids | (Ryrholm et al. 2017) |
| I | Ophioniformes | Mesochorinae | *Mesochorus* (Gravenhorst) | Koinobiont endoparasitoid (obligate hyperparasitism) | 1 | Hyperparasitoid of Hymenoptera, B, I (ao. in Coleoptera, Curcullionidae) | Exposed individuals | Hosts that are (pre)parasitized by other ichneumonids | (Coseglia et al. 1977) |
| I | Ophioniformes | Metopiinae | *Exochus* (Gravenhorst) | Koinobiont endoparasitoid | 1 | Lepidoptera: Tortricidae, Gelechiidae | Larvae in leaves |  | (Georgiev and Kolarov 1999) |
| I | Ophioniformes | Nesomesochorinae | *Nonnus* (Cresson) | Endoparasitoid (ovipositor notch) | 1 | Unknown | Unknown | Likely exposed larvae | (Quicke 2014) |
| I | Ophioniformes | Ophioninae | *Enicospilus* (Stephens) | Koinobiont endoparasitoid | 1 | Lepidoptera: Saturnidae, Noctuidae | Exposed individuals |  | Gauld 1988; Broad and Shaw 2016) |
| I | Ophioniformes | Oxytorinae | *Oxytorus* (Foerster) | Koinobiont endoparasitoid (ovipositor notch) | 1 | Unknown | Unknown | Likely exposed larvae | (Kasparyan and Ruiz-Cancino 2000) |
| I | Ophioniformes | Sisyrostolinae | *Erythrodolius* (Seyrig) | Ectoparasitoid (?) | 1 | Unknown | Unknown |  |  |
| I | Ophioniformes | Stilbopinae | *Stilbops* (Foerster) | Koinobiont endoparasitoid | 1 | Lepidoptera: Incurvariidae | Larvae in leaves | Also eggs | (Quicke 2005) |
| I | Ophioniformes | Tersilochinae | *Neorhacodes* (Hedicke) | Koinobiont endoparasitoid | 1 | Hymenoptera: Crabronidae, Pemphredonidae | Exposed individuals | Eggs/Larvae, exploits existing holes | (Danks 1970; Notton and Shaw 1998; Hansen 2012) |
| I | Ophioniformes | Tersilochinae | *Stethantyx* (Townes) | Koinobiont endoparasitoid | 1 | Coleoptera: Nitidulidae | Bark or Galls |  | (Khalaim and Broad 2013) |
| I | Ophioniformes | Tryphoninae | *Dyspetes* (Foerster) | Koinobiont ectoparasitoid | 1 | Hymenoptera: Symphyta | Exposed individuals | Larvae | (Hinz 1961) |
| I | Ophioniformes | Tryphoninae | *Hercus* (Townes) | Koinobiont ectoparasitoid | 1 | Lepidoptera | Exposed individuals | Larvae | (Bennett 2006) |
| I | Ophioniformes | Tryphoninae | *Idiogramma (*Foerster) | Koinobiont ectoparasitoid |  | Hymenoptera: Xyelidae | Bark or Galls | Larvae concealed in pine cones | (Hilszczañski 1996; van Achterberg and Altenhofer 1997) |
| I | Ophioniformes | Tryphoninae | *Netelia* (Gray) | Koinobiont ectoparasitoid | 1 | Lepidoptera: Noctuidae | Exposed individuals | Larvae | (Shaw 2001; Johns and Whitehouse 2004) |
| I | Ophioniformes | Tryphoninae | *Oedemopsis* (Tschek) | Koinobiont ectoparasitoid | 1 | Lepidoptera: Tortricidae | Larvae in leaves |  | (Loan and Doğanlar 1980) |
| I | Orthopelmatiformes | Orthopelmatinae | *Orthopelma* (Taschenberg) | Koinobiont endoparasitoid | 1 | Hymenoptera: Cynipidae | Larvae in leaves | Also concealed in leaf buds and/or galls, also eggs | (Jones 1983; Bendixen 2026) |
| I | Pimpliformes | Acaenitinae | *Arotes* (Gravenhorst) | Koinobiont endoparasitoid | 1 | Coleoptera: Cerambycidae, Mordellidae | Wood or Mud | Larvae | (Townes and Townes 1960) |
| I | Pimpliformes | Acaenitinae | *Coleocentrus* (Gravenhorst) | Koinobiont endoparasitoid | 1 | Coleoptera: Cerambycidae | Wood or Mud | Larvae | (Naves et al. 2005; Shaw and Lyszkowski 2019) |
| I | Pimpliformes | Collyrinae | *Collyria* (Schiødte) | Koinobiont endoparasitoid | 1 | Hymenoptera: Cephidae | Larvae in leaves | Also concelaed in grass stems, also eggs | (Salt 1932; Streams and Coles 1965) |
| I | Pimpliformes | Cylloceriinae | *Cylloceria* (Schiødte) | Endoparasitoid | 1 | Diptera: Tipulidae, Mycetophilidae | Larvae in loose matter |  | (Wahl 1986) |
| I | Pimpliformes | Diacritinae | *Diacritus* (Vollenhoven) | Unknown | 1 | Unknown | Unknown |  |  |
| I | Pimpliformes | Diplazontinae | *Diplazon* (Viereck) | Koinobiont endoparasitoid | 1 | Diptera: Syrphidae | Exposed individuals | Eggs/Larvae | (Kelly 1914; Schneider 1950) |
| I | Pimpliformes | Orthocentrinae | *Aperileptus* (Foerster) | Koinobiont endoparasitoid | 1 | Diptera: Mycetophilidae, Sciaridae | Larvae in loose matter |  | (Watanabe et al. 2024) |
| I | Pimpliformes | Orthocentrinae | *Eusterinx* (Foerster) | Koinobiont endoparasitoid | 1 | Diptera: Mycetophilidae, Sciaridae | Larvae in loose matter |  | (Fulton 1941; Sivinski 1998) |
| I | Pimpliformes | Pimplinae | *Dolichomitus* (Smith) | Idiobiont ectoparasitoid | 1 | Coleoptera | Exposed individuals | Larvae concealed in wood; access through frass holes | (Kishi 1970) |
| I | Pimpliformes | Pimplinae | *Hymenoepimecis* (Viereck) | Koinobiont ectoparasitoid | 1 | Araneae | Exposed individuals | Spiders | (Eberhard 2000; Sobczak et al. 2009) |
| I | Pimpliformes | Pimplinae | *Pimpla* (Fabricius) | Idiobiont endoparasitoid | 1 | Lepidoptera | Pupae | Also cocoons | (Jackson 1937) |
| I | Pimpliformes | Pimplinae | *Scambus* (Hartig) | Idiobiont ectoparasitoid | 1 | Lepidoptera: Tortricidae | Larvae in leaves |  | (Leius 1967) |
| I | Pimpliformes | Pimplinae | *Theronia* (Holmgren) | Idiobiont endoparasitoid/ectoparasitoid | 1 | Lepidoptera | Pupae | Also silk cocoons | (Townes 1940; Gupta 1983) |
| I | Pimpliformes | Pimplinae | *Tromatobia* (Foerster) | Koinobiont ectoparasitoid | 1 | Araneae | Exposed individuals | Egg cocoons | (Sacher 1988; Rollard 1990) |
| I | Pimpliformes | Poemeniinae | *Poemenia* (Holmgren) | Idiobiont ectoparasitoid | 1 | Hymenoptera: Sphecidae; Coleoptera | Wood or Mud | Larvae concelaed in wood/pine cones | (Townes and Townes 1960; Wahl 2020) |
| I | Pimpliformes | Rhyssinae | *Megarhyssa* (Ashmead) | Idiobiont ectoparasitoid | 1 | Hymenoptera: Siricidae | Wood or Mud | Larvae | (Heatwole et al. 1962, 1964) |
| I | Pimpliformes | Rhyssinae | *Rhyssa* (Gravenhorst) | Idiobiont ectoparasitoid | 1 | Hymenoptera: Siricidae; (Coleoptera, Cerambycidae?) | Wood or Mud | Larvae | (Zondag and Nuttall 1961; Hocking 1968) |
| I | Eucerotinae |  | *Euceros* (Gravenhorst) | Koinobiont ectoparasitoid | 1 | Hyperparasitoid of Hymenoptera in Lepidoptera: Geometridae; Hymenoptera: Diprionidae, *Neodiprion* | Exposed individuals | Larvae, parasitized by other ichneumonids | (Shaw 2014) |
| I | Xoridiformes | Xoridinae | *Odontocolon* (Cushman) | Idiobiont ectoparasitoid | 1 | Coleoptera: Anobiidae, Melandryidae | Wood or Mud | Larvae | (Hoebeke and Mccabe 1977; Cabrera et al. 2002, 2002) |
| I |  | Microleptinae | *Microleptes* (Gravenhorst) | Endoparasitoid | 1 | Diptera: Mycetophilidae, Stratiomyidae | Larvae in loose matter |  | (Wahl 1986; Schwarz 1991) |

**SI Table 2. Species that were initially part of the dataset, but excluded due to missing scale bars for their cross-sections.** This information is presented here to facilitate potential similar studies in the future.

| **Family** | **Higher group** | **Subfamily** | ***Genus*** | **Life history strategy** | **Section source** | **Host group** | **Substrate group** | **Additional info** | **Host source** |
| --- | --- | --- | --- | --- | --- | --- | --- | --- | --- |
| B | Helconoid complex, Macrocentroid subcomplex | Orgilinae | *Stantonia* (Ashmead) | Koinobiont endoparasitoid | 2 | Lepidoptera: Noctuidae, Coleophoridae | Exposed individuals | Larvae accessed through frass holes | (Kula et al. 2010) |
| B | Helconoid complex, Macrocentroid subcomplex | Amicrocentrinae | *Amicrocentrum* (Schulz) | 2 | Lepidoptera: Noctuidae |  | Exposed individuals | Larvae (weakly concealed in stems; access through existing holes) | (van Acherberg 1979; Polaszek 1997) |
| B | Microgastroid complex | Ichneutinae | *Ichneutes* (Nees) | Koinobiont endoparasitoid | 2 | Hymenoptera: Symphyta, Tenthredinidae | Exposed individuals | Eggs/Larvae | (Sharkey and Wharton 1994) |
| B | Euphoroid complex | Euphorinae | *Pygostolus* (Nees) | Koinobiont endoparasitoid | 2 | Coleoptera: Curcullionidae | Exposed individuals | Adults (exposed), larvae (weakly concealed in soil) | (Loan and Holdaway 1961) |
| B | Euphoroid complex | Euphorinae | *Centistes* (Haliday) | Koinobiont endoparasitoid | 2 | Coleoptera: Chrysomelidae | Exposed individuals | Adults | (Shaw 1995a; Schroder and Athanas 2002; Zijp and Blommers 2002; Walsh et al. 2003) |
| B | Euphoroid complex | Cenocoeliinae | *Cenocoelius* (Haliday) | Koinobiont endoparasitoid | 2 | Coleoptera: Cerambicidae, Buprestidae, Scolytidae | Wood or Mud | Larvae | (Saffer 1977; Shaw 1999) |
| B | Helconoid complex | Helconinae | *Helcon* (Nees) | Koinobiont endoparasitoid | 2 | Coleoptera: Cerambicidae, Buprestidae, Curclionidae, Melandryidae | Exposed individuals | Larvae concealed in wood, but probably access through existing holes | (van Achterberg 1975; Shaw and Mendel 2017) |
| B | Cyclostomes | Braconinae | *Iphiaulax* (Foerster) | ectoparasitoid | 2 | Lepidoptera: Pyralidae | Exposed individuals | Larvae (concealed; access through existing holes) | (Wanjala and Khaemba 1987) |

**Phylogenetic tree in Newick format**

(((((((((((((((((((Eusterinx,Aperileptes),(Diacritus,Pimpla)),(((Scambus,Dolichomitus,(Hymenoepimecis,Tromatobia))),(Poemenia,Collyria))),Cylloceria))),((Megarhyssa,Rhyssa),Theronia)),(Diplazon)),(Arotes,Coleocentrus)),(((((Heterischnus,(Carinodes,Ichneumon , Alomya,Holcichneumon)),Adelognathus,Microleptes),(Buathra, Osprynchotus, Xanthocryptus,(Dichrogaster ,Gelis ) )),Agriotypus))),(((((((((((((Enicospilus_1,Enicospilus_2),Nonnus),((Campoplex,Bathyplectes),Diadegma))), (Anomalon,(Heteropelma,Vernamalon))),((Eiphosoma,Pristomerus,Temelucha),(Lycorina))),((Mesoleius,Oetophorus,Pion),(Exhochus)))),(((Cidaphus,Mesochorus),Hybrizon))),(((Glypta,Lissonota, (Banchus,Exetastes)),Stilbops),Oxytorus)),(((Stethantyx,Neorhacodes),Erythrodolius),(Idiogramma,(Hercus,Oedemopsis),Netelia, Dyspetes)))))),(Asperellus )),(Odontocolon)))),(Orthopelma_1,Orthopelma_2)), Euceros),(((((((Campyloneurus,Ligulibracon,Undabracon),(Aleiodes,Clinocentrus),(Odontobracon,Spathius)),(Doryctomorpha,Histeromerus,Thoracoplites))),(Ephedrus,Mesostoa)),(((Austrozele,Macrocentrus),(Microtypus,Stantonia)),(Zele))))));

**References**

Allen T, Kenis M, Norgrove L. 2021. *Eiphosoma laphygmae*, a classical solution for the biocontrol of the fall armyworm, *Spodoptera frugiperda*? J Plant Dis Prot 128:1141–56. https://doi.org/10.1007/s41348-021-00480-9.

Arthur AP, Ewen AB. 1975. Cuticular encystment: a unique and effective defense reaction by cabbage looper larvae against parasitism by *Banchus flavescens* (Hymenoptera: Ichneumonidae). Ann Entomol Soc Am 68:1091–94. https://doi.org/10.1093/aesa/68.6.1091.

Arthur AP, Mason PG. 1985. The life history and immature stages of *Banchus flavescens* (Hymenoptera: Ichneumonidae), a parasitoid of the bertha armyworm, *Mamestra configurata* (Lepidoptera: Noctuidae) in western Canada. Can Entomol 117:1249–55. https://doi.org/10.4039/Ent1171249-10.

Ashley TR, Waddill VH, Mitchell ER, Rye J. 1982. Impact of native parasites on the fall armyworm, *Spodoptera frugiperda* (Lepidoptera: Noctuidae), in South Florida and release of the exotic parasite, *Eiphosoma vitticole* (Hymenoptera: Ichnuemonidae). Environ Entomol 11:833–37.

Barbosa P, Caldas A. 2004. Patterns of parasitoid-host associations in differentially parasitized macrolepidopteran assemblages on Black Willow *Salix nigra* (Marsh) and Box Elder *Acer negundo* L. Basic Appl Ecol 5:75–85. https://doi.org/10.1078/1439-1791-00213.

Barbosa P, Segarra AE, Gross P, Caldas A, Ahlstrom K, Carlson RW, Ferguson DC, Grissell EE, Hodges RW, Marsh PM. 2001. Differential parasitism of macrolepidopteran herbivores on two deciduous tree species. Ecology 82:698–704.

Barron JR. 1997. The Nearctic species of Oetophorus (Hymenoptera, Ichneumonidae). Insecta Mundi 11:117–28. https://digitalcommons.unl.edu/cgi/viewcontent.cgi?article=1261&amp;context=insectamundi.

Bendixen L. 2026. On the biology of two parasitoids of rose gall wasps (*Diplolepis* spp. (Hymenoptera: Diplolepididae)), *Orthopelma mediator* (Thunberg, 1822) and *Orthopelma brevicorne* Morley, 1907 (Hymenoptera: Ichneumonidae: Orthopelmatinae), with a world list of Ichneumonidae known to oviposit into the host egg. Faun Ökol Mitt 13–20. https://doi.org/10.38072/2699-7762/p29.

Bennett AMR. 2006. A new genus of Oedemopsini (Hymenoptera: Ichneumonidae: Tryphoninae) with a key to the genera of the tribe. Can Entomol 138:464–72. https://doi.org/10.4039/n06-807.

Berberet RC. 1982. Effects of host age on embryogenesis and encapsulation of the parasite *Bathyplectes curculionis* in the alfalfa weevil. J Invertebr Pathol 40:359–66. https://doi.org/10.1016/0022-2011(82)90174-4.

Buleza VV. 1990. Search and host choice by *Exetastes cinctipes* Retz. (Hymenoptera: Ichneumonidae) - parasitoid of the genus *Mamestra* (Lepidoptera: Noctuidae). Dokl Biol Sci 314:563–65. https://www.cabidigitallibrary.org/doi/full/10.5555/19941100488.

Cabrera BJ, Marsh PM, Lewis VR, Seybold S. J. 2002. A new species of *Heterospilus* (Hymenoptera: Braconidae) associated with the deathwatch beetle, *Hemicoelus gibbicollis* (LeConte) (Coleoptera: Anobiidae). Pan-Pac entomol 78:7–16. https://archive.org/details/biostor-245432.

Cameron E. 1950. The Biology and economic Importance of *Alomya debellator* (F.), a remarkable Parasite of the Swift Moth, *Hepialus lupulinus* (L.). Bull Entomol Res 41:429–38. https://doi.org/10.1017/S0007485300027723.

Čapek M, van Achterberg C. 1992. A revision of the genus *Microtypus* Ratzeburg (Hymenoptera: Braconidae). Zool Meded 66:323–38. https://repository.naturalis.nl/pub/319175.

Casiraghi M, Andrietti F, Bonasoro F, Martinoli A. 2001. A Note on host detection by *Buathra tarsoleuca* (Schrank) (Hymenoptera: Ichneumonidae), a parasite of *Ammophila sabulosa* (L.) and *Podalonia affinis* (Kirby) (Hymenoptera: Sphecidae). 14:299–312. https://doi.org/10.1023/A:1011163027521.

Chao HF. 1980. Description of *Lycorina spilonotae* Chao with notes on its final-instar larva (Hymenoptera: Ichneumonidae: Lycorininae). Entomotaxonomia 2:165–67.

Chow FJ, Mackauer M. 1986. Host discrimination and larval competition in the aphid parasite *Ephedrus californicus*. Entomol Exp Appl 41:243–54. https://doi.org/10.1111/j.1570-7458.1986.tb00535.x.

Coseglia AF, Simpson RG, Eklund LR. 1977. Biology of *Mesochorus nigripes*: a hyperparasite of *Bathyplectes* Spp. Ann Entomol Soc Am 70:695–98. https://doi.org/10.1093/aesa/70.5.695.

Cossentine JE, Lewis LC. 1987. Development of *Macrocentrus grandii* Goidanich within microsporidian-infected *Ostrinia nubilalis* (Hübner) host larvae. Can J Zool 65:2532–35. https://doi.org/10.1139/z87-382.

Criddle N. 1928. The introduction and establishment of the larch sawfly parasite, *Mesoleius tenthredinis* Morley, into southern Manitoba (Hymen.). Can Entomol 60:51–53. https://doi.org/10.4039/Ent6051-3.

Dancau T, Haye T, Cappuccino N, Mason PG. 2020. Something old, something new: revisiting the diamondback moth (Lepidoptera: Plutellidae) life table after 65 years. Can Entomol 152:70–88. https://doi.org/10.4039/tce.2019.70.

Dangerfield PC, Austin AD. 1998. Biology of *Mesostoa kerri* (Insecta: Hymenoptera: Braconidae: Mesostoinae), an endemic Australian wasp that causes stem galls on *Banksia marginata*. Aust J Bot 46:559–69. https://doi.org/https://doi.org/10.1071/BT97042.

Danks HV. 1970. Biology of some stem‐nesting aculeate Hymenoptera. Trans R Entomol Soc Lond 122:323–99. https://doi.org/10.1111/j.1365-2311.1971.tb00526.x.

Ding D, Swedenborg PD, Jones RL. 1989. Chemical stimuli in host-seeking behavior of *Macrocentrus grandit* (Hymenoptera: Braconidae). Ann Entomol Soc Am 82:232–36.

Dureseau L, Rivet E. 1972. *Ephedrus plagiator*, a parasite of the greenbug in France. J Econ Entomol 65:604–5. https://doi.org/doi.org/10.1093/jee/65.2.604.

Eberhard WG. 2000. The natural history and behavior of *Hymenoepimecis argyraphaga* (Hymenoptera: Ichneumonidae) a parasitoid of *Plesiometa argyra* (Araneae: Tetragnathidae). J Hymenopt Res 9:220–40. https://biostor.org/reference/271.

Elliott JM. 1982. The life cycle and spatial distribution of the aquatic parasitoid *Agriotypus armatus* (Hymenoptera: Agriotypidae) and its caddis host *Silo pallipes* (Trichoptera: Goeridae). J Anim Ecol 51:923. https://doi.org/10.2307/4014.

Fortier JC. 2006. The first host association for *Aleioides pilosus* (=Tetrasphaeropyx Ashmead) (Hymenoptera Braconidae: Rogadinae). Entomol News 117:457–59. https://doi.org/10.3157/0013-872X(2006)117%5B457:TFHAFA%5D2.0.CO;2.

Fortier JC. 2008. The first host association for *Aleiodes shawi* (Hymenoptera: Braconidae: Rogadinae). Entomol News 119:214–16. https://doi.org/10.3157/0013-872X(2008)119%5B214:TFHAFA%5D2.0.CO;2.

Fulton BB. 1941. A luminous fly larva with spider traits (Diptera, Mycetophilidae). Annals of the Entomological Society of America 34:289–302. https://doi.org/10.1093/aesa/34.2.289.

Gauld ID. 1978. A revision of the Anomaloninae (Hymenoptera: Ichneumonidae) Melanesia. I. The genera *Anomalon* Panzer to *Aphanistes* Foerster. Bull Entomol Res 68:501–19. https://doi.org/10.1017/S0007485300009482.

Gauld ID. 1980. A synopsis of the Anomaloninae (Hymenoptera: Ichneumonidae) of sub-Saharan Africa with special reference to species of potential economic importance. Bull Entomol Res 70:33–41. https://doi.org/10.1017/S0007485300009743.

Gauld ID, Wahl DB. 2000. The Labeninae (Hymenoptera: Ichneumonidae): a study in phylogenetic reconstruction and evolutionary biology. Zoological Journal of the Linnean Society 129:271–347. https://doi.org/10.1111/j.1096-3642.2000.tb00015.x.

Georgiev G, Kolarov J. 1999. New Ichneumonidae (Hymenoptera) parasitoids of forest insect pests in Bulgaria. Anz Schadlingskde, Pflanzenschutz, Umweltschutz 72:57–61. https://doi.org/10.1007/BF02770645.

Ghigi A. 1911. Studio sistematico degli Icneumonidi affricani appartenenti al genere *Osprynchotus* Spinola. Annuario del Museo Zoologico della R Università di Napoli (Nuova Serie) 3:14. https://ia801701.us.archive.org/0/items/annuariodelm319091912univ/annuariodelm319091912univ.pdf.

Giraldo-Vanegas H, García R JL. 1994. Comportamiento, descripcion y tiempo de desarrollo de los estados inmadurosde <i>Eiphosoma vitticolle<i> Cresson (Hymenoptera: Ichneumonidae), parasito de *Spodoptera frugiperda* (J. E. Smith) (Lepidoptera: Noctuidae). Agronomía Tropical 44:645–65. http://www.sian.inia.gob.ve/revistas_ci/Agronomia%20Tropical/at4404/Arti/giraldo_h.htm.

Gupta V. 1983. The ichneumonid parasites associated with the gypsy moth (<i>Lymantria dispar>/i>). Contrib Am Entomol Inst 19:1–169. https://biostor.org/reference/286030.

Hansen LO. 2012. The ichneumonid wasp *Neorhacodes enslini* (Ruschka, 1922) (Hymenoptera, Ichneumonidae) in Norway. Nor J Entomol 59:40–42. https://www.researchgate.net/profile/Lars-Hansen-4/publication/260459953_The_ichneumonid_wasp_Neorhacodes_enslini_Ruschka_1922_Hymenoptera_Ichneumonidae_in_Norway/links/5457bc090cf26d5090ab5073/The-ichneumonid-wasp-Neorhacodes-enslini-Ruschka-1922-Hymenoptera-Ichneumonidae-in-Norway.pdf.

Harrison JO. 1963. The natural enemies of some banana insect pests in Costa Rica. J Econ Entomol 56:282–85. https://doi.org/10.1093/jee/56.3.282.

Heatwole H, Davis DM, Wenner AM. 1962. The behaviour of *Megarhyssa*, a genus of parasitic hymenopterans (Ichneumonidae: Ephialtinae). Z Tierpsychol 19:652–64. https://doi.org/10.1111/j.1439-0310.1962.tb00796.x.

Heatwole H, Davis DM, Wenner AM. 1964. Detection of mates and hosts by parasitic insects of the genus *Megarhyssa* (Hymenoptera: lchneumonidae). Am Midl Nat 71:374. https://doi.org/10.2307/2423295.

Hilszczañski J. 1996. The effect of pesticides applied aerially to forest stands on four species of native hymenopterous parasitoids. In: Population Dynamics, Impacts, and Integrated Managment of Forest Defoliating Insects Presented at the Population Dynamics, Impacts, and Integrated Managment of Forest Defoliating Insects. Banská Štiavnica, Slovak Republic: Radnor, PA : USDA Forest Service, Northeastern Research Station. p. 116–21. https://babel.hathitrust.org/cgi/pt?id=umn.31951d01944852x&seq=90.

Hinz R. 1961. Über Blattwespenparasiten (Hym. und Dipt.). Mitt Schweiz entomol Ges. https://doi.org/10.5169/SEALS-401399.

Hinz R, Short J. 1983. Life-history and systematic position of the European *Alomya* species (Hymenoptera: Ichneumonidae). Insect Syst Evol 14:462–66. https://doi.org/10.1163/187631283X00236.

Hocking H. 1968. Studies on the biology of *Rhyssa persuasoria* (l.) (Hymenoptera: Ichneumonidae) incorporating an x‐ray technique. Aust J Entomol 7:1–5. https://doi.org/10.1111/j.1440-6055.1968.tb00690.x.

Hoebeke ER, Mccabe TL. 1977. The life history of *Serropalpus coxalis*, with a description of the larva and pupa (Coleoptera: Melandryidae). Coleopt Bull 31:57–63. https://doi.org/10.5962/p.371930.

Idris AB, Grafius E. 1996. Effects of wild and cultivated host plants on oviposition, survival, and development of diamondback moth (Lepidoptera: Plutellidae) and its parasitoid *Diadegma insulare* (Hymenoptera: Ichneumonidae). Environ Entomol 25:825–33. https://doi.org/10.1093/ee/25.4.825.

Jackson DJ. 1937. Host‐selection in *Pimpla examinator* F. (Hymenoptera). Proceedings of the Royal Entomological Society of London Series A, General Entomology 12:81–91. https://doi.org/10.1111/j.1365-3032.1937.tb00959.x.

Johns CV, Whitehouse MEA. 2004. Mass rearing of two larval parasitoids of *Helicoverpa* spp. (Lepidoptera: Noctuidae): *Netelia producta* (Brullé) and *Heteropelma scaposum* (Morley) (Hymenoptera: Ichneumonidae) for field release. Australian Journal of Entomology 43:83–87. https://doi.org/10.1111/j.1440-6055.2004.00402.x.

Jones D. 1983. The influence of host density and gall shape on the survivorship of *Diastrophus kincaidii* Gill. (Hymenoptera: Cynipidae). Can J Zool 61:2138–42. https://doi.org/10.1139/z83-280.

Kasparyan DR, Ruiz-Cancino E. 2000. First record of the subfamily Oxytorinae (Hymenoptera: Ichneumonidae) in Mexico, and description of new species. Anales del Instituto de Biología, UNAM Serie Zoología 71:125–31. https://revistas.unam.mx/index.php/zoo/article/view/7241.

Kelly EOG. 1914. Notes on the biology of *Diplazon laetatorius* (Fabr.). J Econ Entomol 7:294–97. https://doi.org/10.1093/jee/7.3.294.

Khalaim AI, Broad GR. 2013. Tersilochinae (Hymenoptera: Ichneumonidae) of Costa Rica, part 2. Genera *Megalochus* gen. nov. and *Stethantyx* Townes. Zootaxa 3693. https://doi.org/10.11646/zootaxa.3693.2.8.

Kishi Y. 1970. Difference in the sex ratio of the pine bark weevil parasite, *Dolichomitus sp.* (Hymenoptera: Ichneumonidae), emerging from different host species. Appl entomol Zool 5:126–32. https://doi.org/10.1303/aez.5.126.

Kula RR, Boughton AJ, Pemberton RW. 2010. *Stantonia pallida* (Ashmead) (Hymenoptera: Braconidae) reared from *Neomusotima conspurcatalis* Warren (Lepidoptera: Crambidae), a classical biological control agent of *Lygodium microphyllum* (Cav.) R. Br. (Polypodiales: Lygodiaceae). Proc Entomol Soc Wash 112:61–68. https://doi.org/10.4289/0013-8797-112.1.61.

Leius K. 1967. Food sources and preferences of adults of a parasite, *Scambus buolianae* (Hym.: Ichn.), and their consequences. Can Entomol 99:865–71. https://doi.org/10.4039/Ent99865-8.

Leong JKL, Oatman ER. 1968. The biology of *Campoplex haywardi* (Hymenoptera: Ichneumonidae), a primary parasite of the potato tuberworm. Annals of the Entomological Society of America 61:26–36. https://doi.org/10.1093/aesa/61.1.26.

Loan C, Holdaway FG. 1961. *Pygostolus falcatus* (Nees) (Hymenoptera, Braconidae), a parasite of *Sitona* species (Coleoptera, Curculionidae). Bull Entomol Res 52:473–88. https://doi.org/10.1017/S0007485300055541.

Loan CC, Doğanlar M. 1980. *Oedemopsis scabricula* in British Columbia (Hymenoptera: Ichneumonidae, Tryphoninae). Nat Can 107:11–14. https://www.researchgate.net/publication/258628802_Oedemopsis_scabricula_in_British_Columbia_Hymenoptera_Ichneumonidae_Tryphoninae#fullTextFileContent.

Marsh PM. 1970. The Nearctic Doryctinae, IX. The genus *Odontobracon* and notes on related genera. Pan-Pac Entomol 46:275–83. https://biostor.org/reference/246439.

McGugan BM. 1955. Certain host-parasite relationships involving the spruce budworm. Can Entomol 87:178–87. https://doi.org/10.4039/Ent87178-4.

Miller CA. 1960. The interaction of the spruce budworm, <i<Choristoneura fumiferana</i> (Clem.), and the parasite *Glypta fumiferanae* (Vier.). Can Entomol 92:839–50. https://doi.org/10.4039/Ent92839-11.

Muldrew JA. 1953. The natural immunity of the larch sawfly (*Pristiphora erichsonii* (Htg.)) to the introduced parasite *Mesoleius tenthredinis* Morley, in Manitoba and Saskatchewan. Can J Zool 31:313–32. https://doi.org/10.1139/z53-025.

Murray DA, Kriegel RD, Johnson JW, Howitt AJ. 2017. Natural enemies of cranberry fruitworm, *Acrobasis Vaccinii*, (Lepidoptera: Pyraudae) in Michigan highbush blueberries. Gt Lakes Entomol 29. https://doi.org/10.22543/0090-0222.1905.

Naves P, Kenis M, Sousa E. 2005. Parasitoids associated with *Monochamus galloprovincialis* (Oliv.) (Coleoptera: Cerambycidae) within the pine wilt nematode-affected zone in Portugal. J Pest Sci 78:57–62. https://doi.org/10.1007/s10340-004-0068-z.

Notton DG, Shaw MR. 1998. A review of the Palaearctic Neorhacodinae (Hymenoptera, Ichneumonidae) with *Eremura* Kasparyan, 1995 new to the west Palaearctic. Bull nat Hist Mus Lond (Ent) 67:209–18. https://www.biodiversitylibrary.org/part/76463.

Oatman ER, Platner GR. 1974. The biology of *Temelucha* sp., platensis group (Hymenoptera: Ichneumonidae), a primary parasite of the potato tuberworm. Ann Entomol Soc Am 67:275–80. https://doi.org/10.1093/aesa/67.2.275.

Panteloni RA. 1987. Studi sui parassitoidi (Hym. Ichneu- monidae et Pteromalidae) di *Chrysoperla carnea* (St.) (Neur. Chrysopidae) che ricercano ed aggrediscono gli staid racchiusi nel bozzolo. Boll Ist entomol Guido Grandi Univ studi Bologna 41:241–55. https://www.academia.edu/download/31061930/013_Pantaleoni_1987_ParassBozzChr_.pdf.

Polaszek A. 1997. An overview of parasitoids of African lepidopteran cereal stemborers (Hymenoptera: Chrysidoidea, Ceraphronoidea, Chalcidoidea, Ichneumonoidea, Platygastroidea). International Journal of Tropical Insect Science 17:13–18.

Polaszek A, Fitton MG, Bianchi G, Huddleston T. 1994. The parasitoids of the African white rice borer, *Maliarpha separatella* Ragonot (Lepidoptera: Pyralidae). Bull Entomol Res 84:65–89. https://doi.org/10.1017/S0007485300032247.

Putiler B. 1974. *Hypera postica* and *Bathyplectes curculionis*: Encapsulation of parasite eggs by host larvae in Missouri and Arkansas. Environ Entomol 3:881–82. https://doi.org/10.1093/ee/3.5.881.

Quicke DJ. 2005. Biology and immature stages of *Panteles schnetzeanus* (Hymenoptera: Ichneumonidae), a parasitoid of *Lampronia fuscatella* (Lepidoptera: Incurvariidae). J Nat Hist 39:431–43. https://doi.org/10.1080/00222930410001708669.

Quicke DLJ. 2014. The braconid and ichneumonid parasitoid wasps: biology, systematics, evolution and ecology. 1st ed Wiley. https://doi.org/10.1002/9781118907085.

Rahoo GM, Luff ML. 1987. The biology of *Adelognathus granulatus* Perkins (Hym., Ichneumonidae) a parasitoid of the small gooseberry sawfly, *Pristiphora pallipes* (Lep.) (Hym., Tenthredinidae). J Appl Entomol 104:480–84. https://doi.org/10.1111/j.1439-0418.1987.tb00549.x.

Rollard C. 1990. Approche écobiologique de l’interaction araignée insecte/arachnophage à travers l’exemple d’Argiope bruennichi (Ar., Argiopidae)/Tromatobia ornata (Hym., Ichneumonidae). Bull soc zool Fr 115:379–85.

Rousse P, Villemant C, Seyrig A. 2013. Ichneumonid wasps from Madagascar. VI. The genus *Pristomerus* (Hymenoptera: Ichneumonidae: Cremastinae). EJT. https://doi.org/10.5852/ejt.2013.49.

Ryrholm N, Johansson N, Shaw MR. 2017. The Swedish species of *Cidaphus* (Hymenoptera, Ichneumonidae, Mesochorinae), a new genus and three new species for Sweden. Ent Tidskr 138:203–8. https://docslib.org/doc/3754279/the-swedish-species-of-cidaphus-hymenoptera-ichneumonidae-mesochorinae-a-new-genus-and-three-new-species-for-sweden.

Sacher P. 1988. Eiparasitierung bei *Argiope bruennichi* (Scopoli) durch die schlupfwespe *Tromatobia ornata* Gravenhorst. Presented at the XI Europäisches Arachnologisches Colloquium. Berlin. p. 104–8.

Saffer B. 1977. A systematic revision of the genus *Cenocoelius* (Hymenoptera, Braconidae) in North America, including Mexico. https://www.proquest.com/docview/302859810?pq-origsite=gscholar&fromopenview=true&sourcetype=Dissertations%20&%20Theses.

Salt G. 1932. Superparasitism by *Collyria calcitrator*, Grav. Bull Entomol Res 23:211–16. https://doi.org/10.1017/S0007485300004119.

Santos BF, Aguiar AP. 2013. Phylogeny and revision of *Messatoporus* Cushman (Hymenoptera, Ichneumonidae, Cryptinae), with descriptions of sixty five new species. Zootaxa 3634. https://doi.org/10.11646/zootaxa.3634.1.1.

Saxena AP. 1967. Biology of *Campyloneurus mutator* (Fabricius) (Hymenoptera : Braconidae). Tech Bull Common Inst Biol Control 9:61–72. https://www.cabidigitallibrary.org/doi/full/10.5555/19700500761.

Schneider F. 1950. Die Abwehrreaktion des Insektenblutes und ihre Beeinflussung durch die Parasiten. Vierteljahresschr Naturforsch Ges Zürich 95:22–44. https://kipdf.com/die-abwehrreaktion-des-insektenblutes-und-ihre-beeinflussung-durch-die-parasiten_5add300c7f8b9a66218b460d.html.

Schreurs J. 1965. Investigations on the biology and control of *Glenea lefebtteri*, a noxious longicorn beetle of cacao in West New Guinea. PNG Ag J 17. https://journal.pngagriculture.net/index.php/pngjaff/article/view/513.

Schroder RFW, Athanas MM. 2002. Biological observations of *Centistes gasseni* Shaw (Hymenoptera: Braconidae), a parasitoid of *Diabarotica* spp. (Coleoptera: Chrysomelidae). Proc Entomol Soc Wash 104:554–62. https://biostor.org/reference/55400.

Schwarz M. 1991. Eine neue Art der Gattung *Microleptes* Gravenhorst (Ichneumonidae, Hymenoptera) aus Österreich. Linzer biol Neitr 23:399–405. https://archive.org/details/linzer-lbb-0023-1-0399-0405.

Sharkey MJ, Wharton RA. 1994. A revision of the genera of the world Ichneutinae (Hymenoptera: Braconidae). J Nat Hist 28:873–912. https://doi.org/10.1080/00222939400770471.

Shaw ES. 1995a. A new species of *Centistes* from Brazil (Hymenoptera: Bracnidae: Euphorinae) parasitizing adults of *Diabrotica* (Coleoptera: Chrysomelidae), with key to new worlds species. Proc Entomol Soc Wash 97:153–60. https://ia801508.us.archive.org/10/items/biostor-55864/biostor-55864.pdf.

Shaw MR. 1981. Delayed inhibition of host development by the nonparalyzing venoms of parasitic wasps. Journal of Invertebrate Pathology 37:215–21.

Shaw MR. 1995b. Observations on the adult behaviour and biology of *Histeromerus mystacinus* Wesmael (Hymenoptera: Braconidae). Entomologist 114:1–13. http://www.filming-varwild.com/publications-3.html.

Shaw MR. 1999. Rearing records of two species of *Cenocoelius* Haliday from Britain (Hymenoptera: Braconidae, Cenocoeliinae). Entomol’s Gaz 50:283–86. https://www.researchgate.net/publication/288729017_Rearing_records_of_two_species_of_Cenocoelius_Haliday_from_Britain_Hymenoptera_Braconidae_Cenocoeliinae.

Shaw MR. 2001. Interactions between adults of some species of *Netelia* Gray (Hymenoptera: Ichneumonidae: Tryphoninae) and their caterpillar hosts (Lepidoptera). J Hymenopt Res 10:101–11. https://biostor.org/reference/300.

Shaw MR. 2014. Illustrated notes on the biology of two European species of *Euceros* Gravenhorst (Hymenoptera: Ichneumonidae: Eucerotinae). Proceedings of the Russian Entomological Society 85:122–32. https://doi.org/10.47640/1605-7678_2014_85_1_122.

Shaw MR, Aeschlimann J-P. 1994. Host ranges of parasitoids (Hymenoptera: Braconidae and Ichneumonidae) reared from *Epermenia chaerophyllella* (Goeze) (Lepidoptera: Epermeniidae) in Britain, with description of a new species of *Triclistus* (Ichneumonidae). J Nat Hist 28:619–29. https://doi.org/10.1080/00222939400770281.

Shaw MR, Lyszkowski RM. 2019. A second British specimen of *Coleocentrus excitator* (Poda) (Hymenoptera: Ichneumonidae, Acaentinae) from Scotland. Br J Ent Nat Hist 32:327–29. http://filming-varwild.com/publications-3.html.

Shaw MR, Mendel H. 2017. A rearing of *Helcon claviventris* Wesmael, a rare British braconid wasp (Hymenoptera: Braconidae: Helconinae), with advice on labelling substrate rearings. Br J Ent Nat Hist 30:75–78. www.filming-varwild.com/publications-3.html.

Sivinski JM. 1998. Phototropism, Bioluminescence, and the Diptera. Fla Entomol 81:282–92. https://doi.org/10.2307/3495919.

Sobczak JF, Loffredo APS, Penteado-Dias AM, Gonzaga MO. 2009. Two new species of *Hymenoepimecis* (Hymenoptera: Ichneumonidae: Pimplinae) with notes on their spider hosts and behaviour manipulation. Journal of Natural History 43:2691–99. https://doi.org/10.1080/00222930903244010.

Sonan J. 1930. A few Host-known Ichneumonidae found in Formosa (Hym.) (2). Trans Nat Hist Soc Formosa 20:137–44. https://www.cabidigitallibrary.org/doi/full/10.5555/19300501309.

Stairs G. 1983. Oviposition by *Glypta fumiferanae* (Hymenoptera: Ichneumonidae) in *Choristoneura fumiferana* (Lepidoptera: Tortricidae) larvae and the effect of honey on their fecundity and survival. Can Entomol 115:689–92.

Streams FA, Coles LW. 1965. Wheat stem sawfly parasites, *Collyria calcitrator* and *Pediobius nigritarsis*, in eastern United States. J Econ Entomol 58:303–6. https://doi.org/10.1093/jee/58.2.303.

Thomas JA, Elmes GW. 1993. Specialized searching and the hostile use of allomones by a parasitoid whose host, the butterfly *Maculinea rebeli*, inhabits ant nests. Anim Behav 45:593–602. https://doi.org/10.1006/anbe.1993.1069.

Townes H, Townes M. 1960. Ichneumon-flies of America North of Mexico pt. 2: Subfamilies Ephialtinae, Xoridinae, and Acaenitinae. Bulletin of the United States National Museum 1–676. http://hdl.handle.net/10088/10225.

Townes HK. 1940. A revision of the Pimplini of Eastern North America (Hymenoptera, Ichneumonidae). Annals of the Entomological Society of America 33:283–323. https://doi.org/10.1093/aesa/33.2.283.

Valentine EW. 1967. A list of entomophagous insects of New Zealand. N Z Jl Sci 10:1100–1210. https://bugz.ento.org.nz/detail/e11044a3-1647-45d1-a21a-4d7c3c510d4b.

van Acherberg C. 1979. A revision of the species of Amicrocentrinae, a new subfamily (Hymenoptera, Braconidae) with a description of the final larval instar of *Amicrocentrum curvinervis* by J. R. T. Short. Tijdschr Entomol 122:1–28. https://biostor.org/reference/49891.

van Achterberg C. 1975. A revision of the tribus *Blacini* (Hymenoptera, Braconidae, Helconinae). Tijdschr Entomol 118:159–322. https://archive.org/details/biostor-49868/page/174/mode/2up.

van Achterberg C. 1979. A revision of the subfamily Zelinae auct. (Hymenoptera, Braconidae). Tijdschr Entomol 122:241–479. https://www.biodiversitylibrary.org/part/66354.

van Achterberg C. 1987. Revisionary notes on the Macrocentrinae from far east USSR (Hymenoptera: Braconidae). Zool Meded 61:243–62. https://repository.naturalis.nl/pub/318072.

van Achterberg C. 1993. Revision of the subfamily Macrocentrinae Foerster (Hymenoptera: Braconidae) from the Palaearctic region. Zool Verh, Zoologische verhandelingen 286:1–105. https://repository.naturalis.nl/pub/317629.

van Achterberg C, Altenhofer E. 1997. *Xyeloblacus* gen. nov. (Hymenoptera: Braconidae: Blacinae) parasitoid of Xyelinae (Xyelidae: Hymenoptera). Zool Med 71:291–98.

van Achterberg K, Durán JM. 2011. Oviposition behaviour of four ant parasitoids (Hymenoptera, Braconidae, Euphorinae, Neoneurini and Ichneumonidae, Hybrizontinae), with the description of three new European species. ZooKeys 125:59–106. https://doi.org/10.3897/zookeys.125.1754.

van Baarlen P, Topping CJ, Sunderland KD. 1996. Host location by *Gelis festinans*, an eggsac parasitoid of the linyphiid spider *Erigone atra*. Entomologia Exp Applicata 81:155–63. https://doi.org/10.1111/j.1570-7458.1996.tb02027.x.

Wahl DB. 1986. Larval structures of oxytorines and their significance for the higher classification of some Ichneumonidae (Hymenoptera). Syst Entomol 11:117–27. https://doi.org/10.1111/j.1365-3113.1986.tb00171.x.

Wahl DB. 2020. The mature larva of *Poemenia albipes* Cresson (Hymenoptera: Ichneumonidae, Poemeniinae) and a review of the biology of Nearctic *Poemenia*. Zootaxa 4743. https://doi.org/10.11646/zootaxa.4743.1.11.

Wahl DB, Green N. 2020. *Stenarella domator* (Poda) [Hymenoptera: Ichneumonidae], a newly introduced species to the Nearctic, and a summary of adventive Nearctic species of Ichneumonidae. Zootaxa 4743. https://doi.org/10.11646/zootaxa.4743.1.8.

Walsh GC, Athanas MM, Salles LAB, Schroder RFW. 2003. Distribution, host range, and climatic constraints on *Centistes gasseni* (Hymenoptera: Braconidae), a South American parasitoid of cucumber beetles, *Diabrotica* spp. (Coleoptera: Chrysomelidae). Bull Entomol Res 93:561–67. https://doi.org/10.1079/BER2003263.

Wang Q, Shi G. 2001. Host preference and sex allocation of three hymenopteran parasitoid species (Ichneumonidae and Braconidae) of a longicorn pest, *Oemona hirta* (Fabr.) (Col., Cerambycidae). J Applied Entomology 125:463–67. https://doi.org/10.1046/j.1439-0418.2001.00582.x.

Wang X-Y, Yang Z-Q, Gould JR, Wu H, Ma J-H. 2010. Host-seeking behavior and parasitism by *Spathius agrili* Yang (Hymenoptera: Braconidae), a parasitoid of the emerald ash borer. Biol Control 52:24–29. https://doi.org/10.1016/j.biocontrol.2009.09.008.

Wanjala FME, Khaemba BM. 1987. The biology and behaviour of *Iphiaulax varipalpis* [Hymenoptera: Braconidae] as a parasite of *Dirphya nigricornis* [Coleoptera: Cerambycidae]. Entomophaga 32:281–89. https://doi.org/10.1007/BF02373252.

Watanabe K, Mukai H, Sueyoshi M. 2024. Review of the ichneumonid parasitoids of the fungus gnats infesting edible fungi in Japan, with a new species of *Orthocentrus* Gravenhorst, 1829. JPN J Syst Entomol 30:182–95. https://doi.org/https://doi.org/10.69343/jjsystent.30.2_182.

Weseloh RM. 1986. Hyperparasites of the gypsy moth (Lepidoptera: Lymantriidae): Field attack patterns on *Cotesia melanoscela* (Hymenoptera: Braconidae) at different host densities and on different-sized host clumps. Ann Entomol Soc Am 79:308–11. https://doi.org/10.1093/aesa/79.2.308.

Yang Z-Q, Wang X-Y, Gould JR, Reardon RC, Zhang Y-N, Liu G-J, Liu E-S. 2010. Biology and behavior of *Spathius agrili*, a parasitoid of the emerald ash borer, *Agrilus planipennis*, in China. J Insect Sci 10:1–13. https://doi.org/10.1673/031.010.3001.

Zijp J, Blommers LHM. 2002. Biology of *Centistes delusorius*, a parasitoid of adult apple blossom weevil. Agr Forest Entomol 4:275–82. https://doi.org/10.1046/j.1461-9563.2002.00148.x.

Zondag R, Nuttall MJ. 1961. *Rhyssa lineolata* (Kirby) (Hymenoptera: Ichneumonidae: Pimplinae) A species new to New Zealand. N Z Entomol 2:40–44. https://doi.org/10.1080/00779962.1961.9722800.

Zong S-X, Sheng M-L, Luo Y-Q, Lu C-K. 2012. *Lissonota holcocerica* Sheng sp.n (Hymenoptera: Ichneumonidae) parasitizing *Holcocerus hippophaecolus* (Lepidoptera: Cossidae) from China. Journal of Insect Science 12:1–7. https://doi.org/10.1673/031.012.11201.
